# Convergence and divergence of genetic and modular networks between diabetes and breast cancer

**DOI:** 10.1111/jcmm.12504

**Published:** 2015-03-06

**Authors:** Xiaoxu Zhang, Yingying Zhang, Yanan Yu, Jun Liu, Ye Yuan, Yijun Zhao, Haixia Li, Jie Wang, Zhong Wang

**Affiliations:** a Institute of Basic Research in Clinical Medicine, China Academy of Chinese Medical SciencesBeijing, China; b Nanjing University of Chinese MedicineNanjing, China; c China Academy of Chinese Medical SciencesBeijing, China; d Department of Cardiology, Guang'anmen Hospital, China Academy of Chinese Medical SciencesXicheng District, Beijing, China

**Keywords:** breast cancer, diabetes, type 2 diabetes, gene interaction network, modularized analysis

## Abstract

Diabetes mellitus (DM) and breast cancer (BC) can simultaneously occur in the same patient populations, but the molecular relationship between them remains unknown. In this study, we constructed genetic networks and used modularized analysis approaches to investigate the multi-dimensional characteristics of two diseases and one disease subtype. A text search engine (Agilent Literature Search 2.71) and MCODE software were applied to validate potential subnetworks and to divide the modules, respectively. A total of 793 DM-related genes, 386 type 2 diabetes (T2DM) genes and 873 BC-related genes were identified from the Online Mendelian Inheritance in Man database. For DM and BC, a total of 99 overlapping genes, 9 modules, 29 biological processes and 7 pathways were identified. Meanwhile, for T2DM and BC, 56 overlapping genes, 5 modules, 20 biological processes and 12 pathways were identified. Based on the Gene Ontology functional enrichment analysis of the top 10 non-overlapping modules of the two diseases, 10 biological functions and 5 pathways overlapped between them. The glycosphingolipid and lysosome pathways verified molecular mechanisms of cell death related to both DM and BC. We also identified new biological functions of dopamine receptors and four signalling pathways (Parkinson's disease, Alzheimer's disease, Huntington's disease and long-term depression) related to both diseases; these warrant further investigation. Our results illustrate the landscape of the novel molecular substructures between DM and BC, which may support a new model for complex disease classification and rational therapies for multiple diseases.

## Introduction

Diabetes mellitus (DM) and breast cancer (BC) are common diseases with high morbidity that are increasingly recognized to occur in the same patient populations. Researchers have noted common factors between the two diseases, including factors related to pathological processes, such as family history, obesity and alcohol intake 1,2. In the current study, we have also identified intersection points during pathogenesis and in pathologic mechanisms and pharmacokinetics. One meta-analysis has suggested that women with type 2 diabetes (T2DM) might be at increased risk of BC 3. Meanwhile, the analysis of population-based medical records in Ontario, Canada, has indicated that postmenopausal women with BC are more likely to develop DM than women without BC 4. The Increased weight gain of adjuvant chemotherapy or oestrogen suppression may further promote diabetes 5. A high body mass index (BMI) is a risk factor for insulin resistance 3. In post-menopausal populations, obesity have been established as a risk factor for BC 6,7. A study showed that insulin has the ability to stimulate aromatase activity further increasing the levels of bioavailable estradiol, which suggested that insulin could also have indirect effects on BC progression 8. Insulin resistance predisposes DM and insulin resistance of type 2 diabetes lead to the increased risk of BC 9.

Researchers have attempted to determine the mechanisms related to these associations. Oxidative stress and inflammatory state associated with an increase in bodyweight are common to both T2DM and BC 1, and some studies have focused on the relationship between T2DM and BC 10,11. A study hypothesized three mechanisms that were the relationship between DM and BC including activation of the insulin-like-growth-factor pathway, activation of the insulin pathway and regulation of endogenous sex hormones 12. However, the molecular mechanisms between the two diseases still need to further explore from the system aspect. In this study, we created a modular network representation of T2DM, a subtype of DM, to explore the relationship between DM (and T2DM) and BC at the molecular level and we made detailed observations of the common features of these two diseases.

Despite the numerous treatment options for diabetes, individualized long-term treatment remains challenging in many cases 13,14. For BC, the understanding of its initiation and progression, as well as its identification, also remain challenging 15,16.

Gene-based network analysis is a promising method for systems biology studies that takes full advantage of large online databases. This approach can be used to map genes, pathways and diseases into networks showing activation, inhibition and combinations of connections 17. Recent evidence has indicated that modular structures may exist in biological systematic networks 18, and researchers continue to study the correlations among diseases from the perspective of the network module 19.

Therefore, in this study, we used target genes to construct a pathological molecular network to explore the complicated relationships between DM (T2DM) and BC, and we aimed to reveal their relationship from the perspective of a system network module.

## Materials and methods

### Network construction and analysis

Genes related to BC and diabetes were derived from Online Mendelian Inheritance in Man (OMIM), a database of human genes and genetic disorders. The results were entered into the Agilent Literature Search software v. 2.71, which was used to construct a network representation of gene/protein associations. Finally, Cytoscape software was used to visualize this network and to analyse the topology of this network. For instance, network parameters such as clustering coefficient, network diameter, network centralization and network radius were calculated and illustrated.

### Identification of modules

We used MCODE, a clustering algorithm-based software, as a discovery tool in network module division, and the results were visualized using Cytoscape software (parameters: connectivity threshold = 2, core threshold K = 2 and node score threshold = 0.2).

### Functional enrichment analysis

Hypergeometric distribution tests were performed to analyse the function of the modules using DAVID software (http://david.abcc.ncifcrf.gov/) (parameters: count = 2, EASE = 0.01, species and background = *Homo sapiens*). Using Gene Ontology (GO) and Kyoto Encyclopedia of Genes and Genomes (KEGG) annotation, the biological processes and KEGG pathway corresponding to the modules were identified, and the P values were ranked.

## Results

### DM- and BC-related genes in the OMIM database

**Figure 1:**
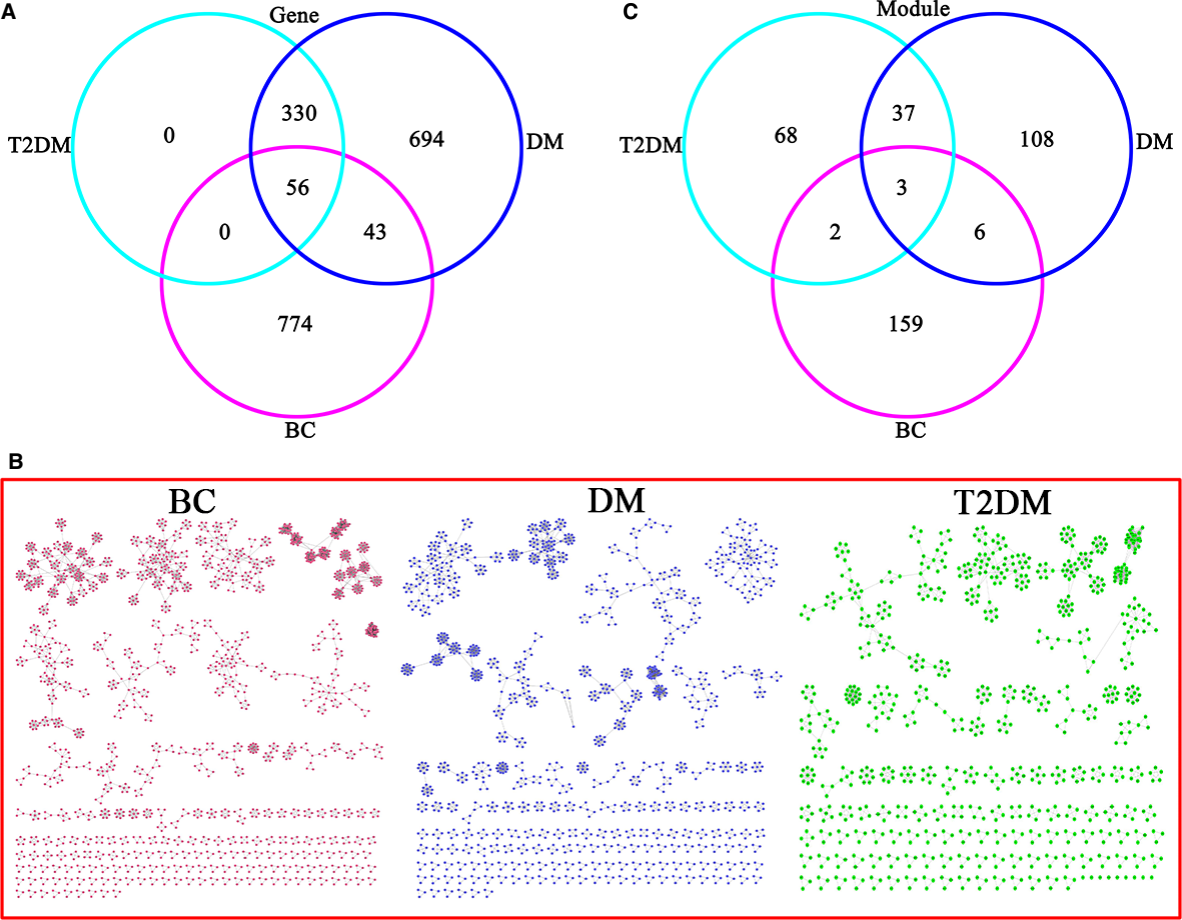
Module networks for BC, DM and T2DM. (A) The 56 overlapping genes among BC, DM and T2DM. (B) BC-, DM- and T2DM-related module networks. (C) The three overlapping modules among BC, DM and T2DM.

Our search of the OMIM database revealed 793 DM-related, 386 T2DM and 873 BC-related genes (Table S1). A total of 56 overlapping genes were detected between the three diseases, and these overlapping genes accounted for 7.06% (56/793) of the known DM-related genes, 14.51% (56/386) of T2DM-related genes and 6.41% (56/873) of BC-related genes (Fig.1A).

### Disease network property analysis

**Figure 2:**
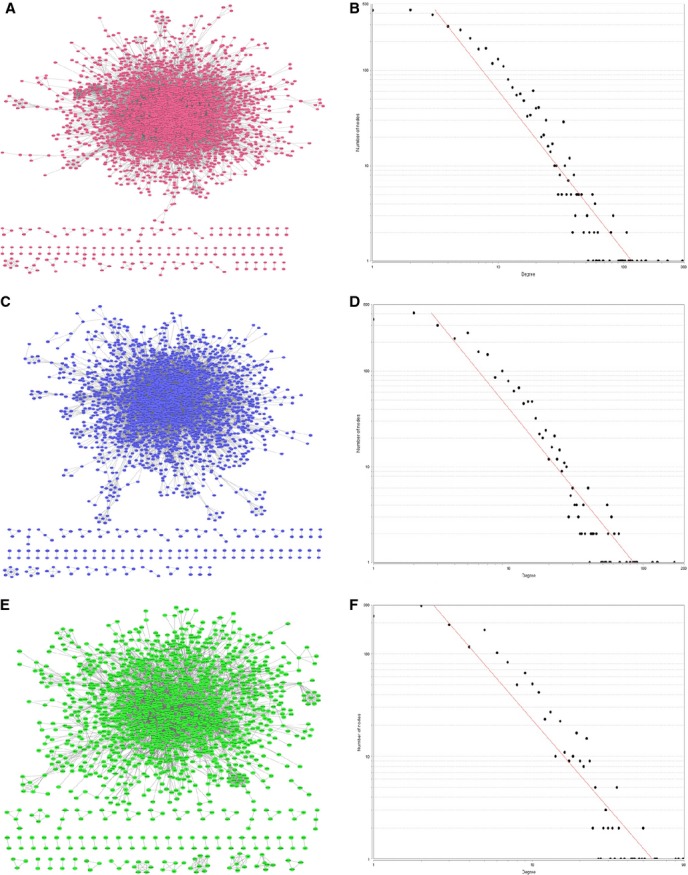
Characteristics of the disease-related gene interaction network. (A) BC-related gene interaction network. (C) DM-related gene interaction network and (E) T2DM-related gene interaction network. The edge in the network is shown as the interaction between two genes. Node degree and betweenness of the (B) BC, (D) DM and (F) T2DM networks from the Centiscape Scatter Plot are similar. The three gene interaction networks are scale-free. The number of nodes increases sharply when the node degree is greater than or equal to 10 in the three networks. Horizontal axis indicates betweenness; vertical axis shows degree; and each graph represents nodes in the networks.

Using Agilent Literature Search v. 2.71 software, respective networks were created. The network for DM-related genes included 2654 nodes (genes) and 10,104 edges (interactions). The network for T2DM-related genes included 1613 nodes (genes) and 5181 edges (interactions), and the network for BC-related genes included 3540 nodes (genes) and 16,545 edges (interactions; Fig.2A, C and E). The topological attributes and modularity of the networks for these three networks are listed in Table1. Similar distributions of node degree that followed the power-law distribution appeared in all of the networks. The three gene interaction networks were scale-free networks (Fig.2B, D and F).

**Table 1:** Comparison of the network topological attributes and modularity of the BC-, DM- and T2DM-related networks

	DM	BC	T2DM
Clustering coefficient	0.591	0.569	0.603
Network diameter	11	10	11
Network radius	1	1	1
Network centralization	0.061	0.079	0.051
No. of nodes	2654	3540	1613
Edges	10104	16545	5181
Genes	793	873	386
Percentage of overlapping genes to total value	7.06% (56/793)	6.41% (56/873)	14.51%(56/386)
Clusters	154	170	110
Average size	9.221	11.035	7.536
Maximum size	133	227	95
Minimum size	3	3	3
Modularity	0.323	0.291	0.385
Percentage of overlapping module of total module value	1.94% (3/154)	1.76% (3/170)	2.72% (3/110)

### Modularity analysis

**Figure 3:**
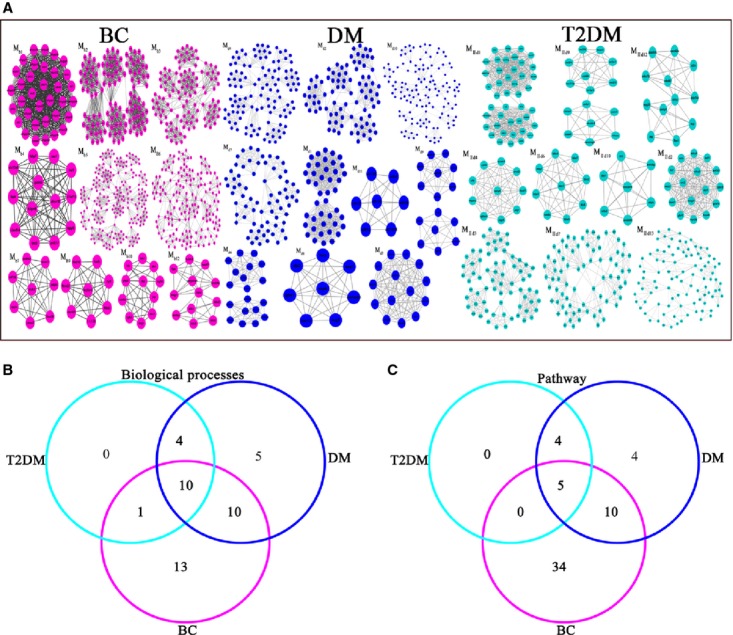
Functional annotation of the top 10 non-overlapping modules. (A) The top 10 non-overlapping module networks for BC, DM and T2DM. (B) 620 biological functions of BC were divided into 34 categories; 335 biological functions of DM were divided into 29 categories; and 114 biological functions of T2DM were divided into 15 categories. (C) 61 BC pathways were identified (divided into 49 categories), 23 pathways of DM were identified and 9 pathways of T2DM were identified.

Entering these results into MCODE v1.13 software yielded 170, modules from the BC network, 154 modules from the DM network and 110 modules from the T2DM network (Fig.1B and C). Nine overlapping modules were identified between DM and BC: M_b11.d12.2d11_, M_b26.d32_, Mb_52.d50_, M_b70.d67_, M_b105.d89.2d68_, M_b136.d107_, M_b153.d141.2d99_, M_b157.d147_ and M_b159.d146_. Five overlapping modules were identified between T2DM and BC: M_b8.2d8_, M_b11.d12.2d11_, M_b105.d89.2d68_, M_b139.2d78_ and M_b153.d141.2d99_. Forty overlapping modules were identified between DM and T2DM: M_2d5.d5_, M_2d16.d21_, M_2d17.d20_, M_2d20.d17_, M_b11.d12.2d11_, *etc*. The first ten non-overlapping modules of the BC, DM and T2DM networks sorted by MCODE score are shown in Figure3A.

## Functional enrichment analysis

### Common modules between BC and DM

**Figure 4:**
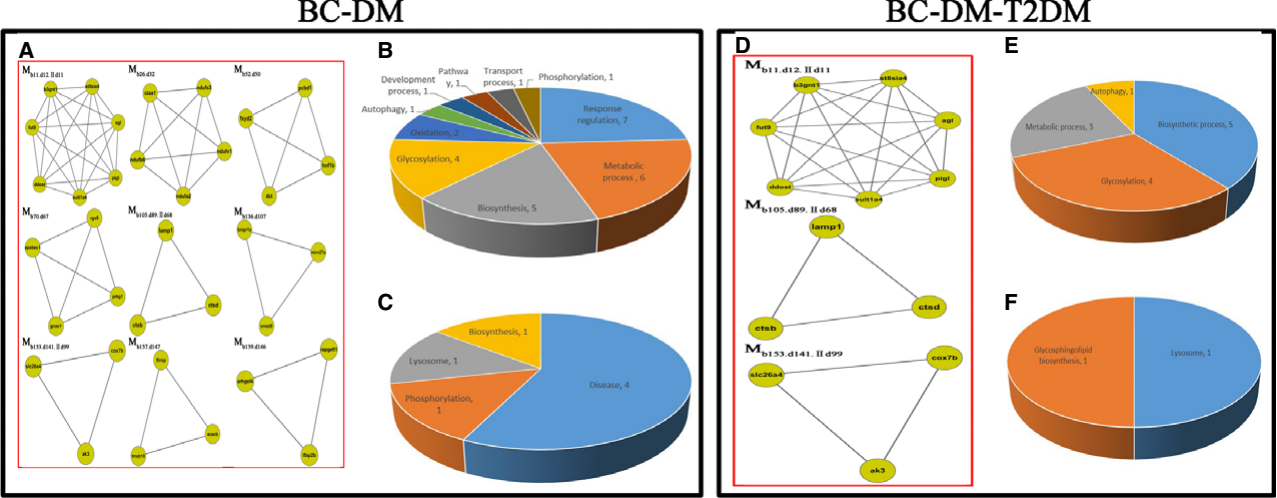
(A) BC and DM shared 9 overlapping modules. (B) Biological functions of BC and DM shared 9 overlapping modules. (C) Pathways of BC and DM shared 9 overlapping modules. (D) BC, DM and T2DM shared 3 overlapping modules. (E) Biological functions of BC, DM and T2DM shared 3 overlapping modules. (F) Pathways of BC, DM and T2DM shared 3 overlapping modules.

The BC and DM networks shared 9 overlapping modules (Fig.4A) with 29 biological functional annotations (Fig.4B), including 7 for response processes, 6 for metabolic processes, 5 for biosynthesis, 4 for glycosylation and 2 each for oxidation, autophagy, development processes, pathways, transport processes and phosphorylation. Seven pathways were identified, including 1 each for disease-related, biosynthesis, lysosome and phosphorylation pathways (Fig.4C).

### Common modules among BC, DM and T2DM networks

**Figure 5:**
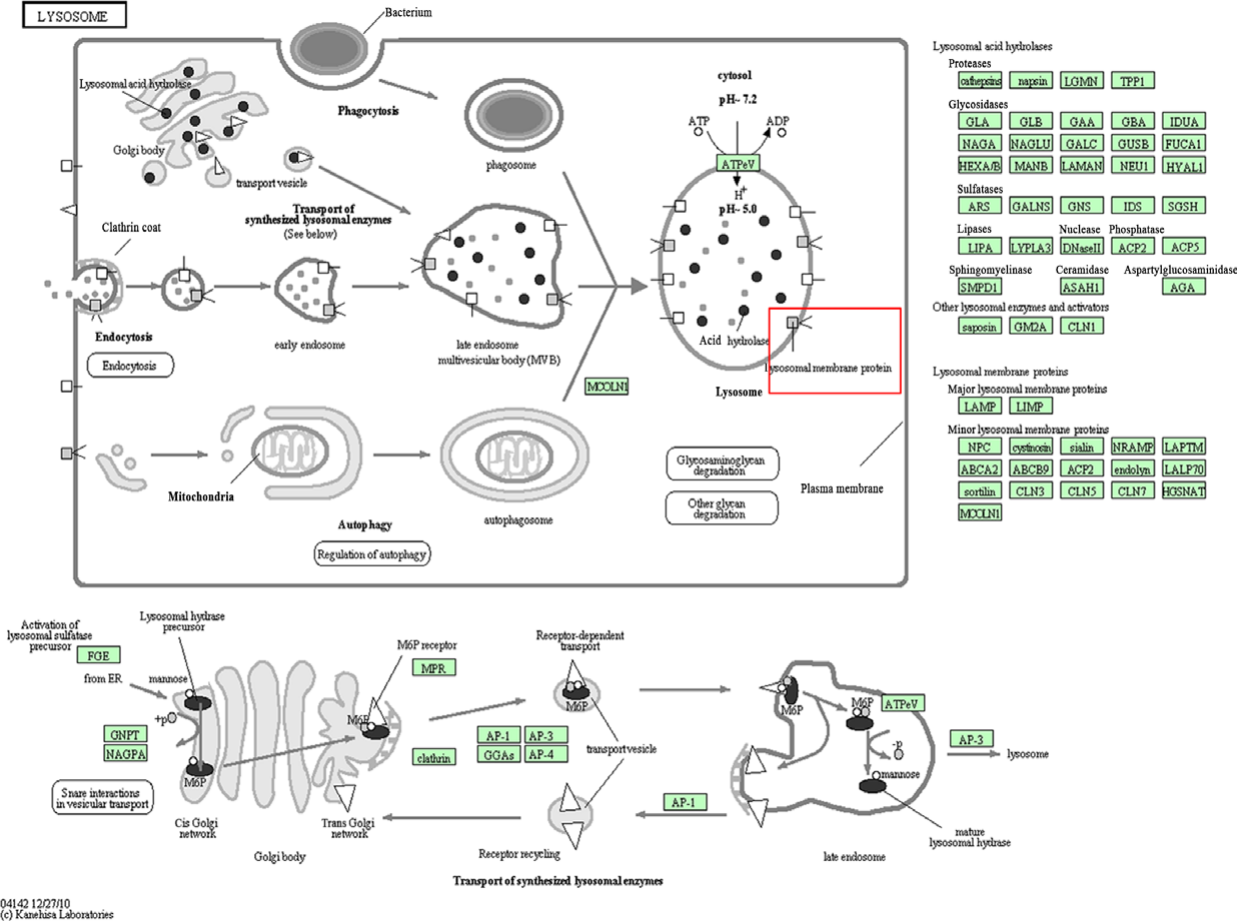
Overview of the Lysosome pathway. The module appears in this pathway marked with a red frame.

The BC, DM and T2DM networks shared 3 overlapping modules (Fig.4D) with 13 biological functional annotations (Fig.4E), including 5 for biosynthetic process, 4 for glycosylation, 3 for metabolic processes and 1 for autophagy. Two overlapping pathways were identified (Fig.4F): lysosome (Fig.5) and glycosphingolipid (GSL) biosynthesis.

### Common modules between BC and T2DM networks

**Figure 6:**
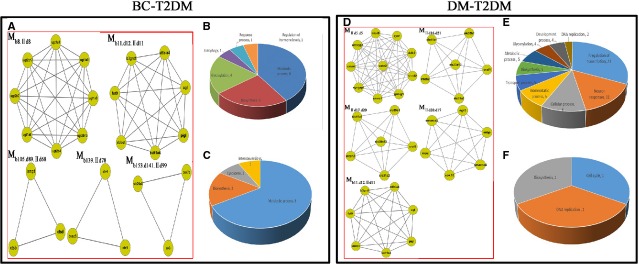
(A) BC and T2DM shared 5 overlapping modules. (B) Biological functions of BC and DM shared 5 overlapping modules. (C) Pathways of BC and DM shared 5 overlapping modules. (D) DM and T2DM shared 40 overlapping modules (The top 5 modules are list in). (E) Biological functions of DM and T2DM shared 40 overlapping modules (The top 5 modules are list in). (F) Pathways of DM and T2DM shared 40 overlapping modules (The top 5 modules are list in).

The BC and T2DM networks shared 5 overlapping modules (Fig.6A) with 20 biological functional annotations (Fig.6B), including 8 for metabolic processes, 5 for biosynthesis and 1 each for glycosylation, autophagy, response processes regulation of hormone levels. Twelve common pathways were identified (Fig.6C), including 8 for metabolic processes, 2 for biosynthesis and 1 each for lysosomes and interconversions.

### Common modules between DM and T2DM networks

The DM and T2DM networks shared 40 overlapping modules; the first 5 modules are listed in Figure6D. These included 73 biological functional annotations (Fig.6E), such as transcription processes, neuron processes and cellular processes. Three common pathways were identified: cell cycle, DNA replication and glycosphingolipid biosynthesis (Fig.6F).

### Unique modules for different diseases

On the basis of the GO functional enrichment analysis of the top 10 non-overlapping modules of the two diseases, as sorted by MCODE score, we identified 335 GO biological annotations and 23 pathways from modules in the DM network; 620 GO biological annotations and 61 pathways from modules in the BC network; and 114 GO biological annotations and 9 pathways from modules in the T2DM network (Fig.3B and C). The top 10 non-overlapping modules according to specific functional enrichment analysis are listed in Table S2.

## Discussion

Many common biological backgrounds between DM and BC were revealed in our study. DM and BC share 99 overlapping genes that may be responsible for the genetic similarity of the two diseases. For example, insulin-like growth factor (IGF) 1R overexpression is associated with DM development 20. Hyperglycaemia promotes BC development by altering IGF1R 21. After a diagnosis of BC, women with the BRCA2 mutation face a 2-fold increase in the risk of DM, which is exacerbated by a high BMI 22. A previous study indicated that women who carry BRCA2 mutations have a substantially increased risk of developing BC and DM 22,23. The 56 overlapping genes indicate a certain degree of genetic similarity among BC, DM and T2DM. For example, BRCA2, CYP19A1, IGF1, IGF1R, FANCA and HNF1A have been identified as biomarkers or therapeutic targets of the two diseases based on direct evidence from the Comparative Toxicogenomics Database (http://cdiabetesbase.org/).

Previous studies have demonstrated that glycation and oxidation of High Density Lipoprotein in diabetic patients could lead to abnormal actions on MDA-MB-231 cell proliferation, migration and invasion, thereby promoting the progression of BC 10. Individuals with obesity, the metabolic syndrome and T2DM have increased BC incidence and mortality 24–26. Additionally, phosphoinositide phosphatases are implicated in a large and diverse array of human diseases, including cancer and diabetes 27. Observational studies have shown that the daily use of metformin in BC patients imparts a survival benefit and a higher pathologic complete response after neoadjuvant chemotherapy 28,29. One meta-analysis has suggested that metformin is associated with a reduced risk of cancer in diabetic patients 30. In addition, impaired autophagy has been associated with the pathogenesis of BC and diabetes 31. Furthermore, otelixizumab, a chimeric CD3 antibody, has been genetically engineered to remove the glycosylation site in the Fc domain to treat diabetes 32. This body of research further confirms correlations between DM and BC.

In this study, DM and BC were found to share two overlapping pathways: GSL and lysosome biosynthesis. GSLs might have an effect on signal transduction related to insulin receptors and on epidermal growth factor receptors that are important for diabetes 33. GSLs are especially concentrated in plasma membrane lipid domains that are specialized for cell signalling. Plasma membranes have typical structures called rafts and caveola domain structures, with large amounts of sphingolipids, cholesterol and sphingomyelin 33. Sphingolipids exert biological effects through cellular proliferation, differentiation and cell death, and they interact with several pathways involved in insulin resistance, oxidative stress, inflammation and apoptosis, all of which are linked to T2DM 34–36. Studies have illustrated aberrant glycosylation in BC cells, and glycosylation changes are associated with GSLs 37,38. Ceramide glycosylation, which is overexpressed in metastatic breast tumours controlling GSL synthesis, plays a role in modulating BC stem cells 39–41. BC cells affect proteins made downstream of lysosomal production, activating cell death 42. Insulin-like growth factors play a critical role in BC, *i.e*. down-regulation *via* a lysosome-dependent pathway 43–45. Autophagosomes are responsible for cell death and delivering extra proteins to lysosomes for recycling in the pathogeneses of cancer and diabetes. 46. Thus, two common pathways are implicated in the cell death mechanism of these two linked diseases 47,48. Our analysis of the non-overlapping network, modules revealed common biological processes related to the two diseases, for example, pathogenesis of metabolic processes 49–51. In contrast, the non-overlapping modules are related to some unique biological processes related to the specific disease. For example, a study found that patients with DM may develop corneal complications and delayed wound healing 52. Chromosome localization as a biological process has been associated with risk of BC in a recent genome-wide association study of women of European ancestry 53. These non-intersecting biological processes further validate the feasibility of modularized network analysis approaches.

The construction of molecular networks and the function of the module annotation enable us to systematically scan the overall picture and the details of these two linked diseases. Using system analysis, we found that some new overlapping biological processes and signalling pathways, for example, the dopamine receptor signalling pathway and Parkinson's disease, Alzheimer's disease, Huntington's disease and long-term depression. Modularized network analyses provide us with new knowledge and direction for future studies. Of course, real-time updating of online databases requires that we update our analyses as these databases expand in the future.

## Conclusion

The overlapping modules of function annotation between DM and BC were investigated in this study. Based on overlapping genes, network modules were established to identify common pathogeneses between DM and BC. In addition to our gene analysis, a large body of literature was reviewed to validate the dependent and independent risk factors that co-exist between BC and DM. We also identified some new biological functions and signalling pathways related to the two diseases that need to be explored further, for example, dopamine receptor biological functions and Parkinson's disease, Alzheimer's disease, Huntington's disease and long-term depression. Our study illuminates the landscape of the genetic relationship between DM and BC, which may provide a new foundation for drug development and clinical medicine.

## Conflicts of interest

The authors declare no conflict of interest.

## Supporting Information

**Data S1** DM- and BC-related genes in the OMIM database.
