# Supplementary material for: Convergence and divergence of genetic and modular networks between diabetes and breast cancer
Source: J Cell Mol Med. 2015 Mar 6;19(5):1094–102. doi: 10.1111/jcmm.12504 (PMC4420611; doi:10.1111/jcmm.12504)
Supplement: Supplementary file 1 [file jcmm0019-1094-sd1.doc]

**Supplemental Table 1**

**BC,DM and T2DM-related genes**

| **Disease** | **Gene** |
| --- | --- |
| DM | AAA1,STAT3,DPP4,GNAS,AKR1B1,GANC,ADRA2A,APOE,APP,APCS,ANIB1,AGT,ACE,PAX6,CD59,APOA1,AQP2,CYP19A1,ARRB2,ATXN3,ADRB2,ADRB3,HTR1A,ABO,SLC14A1,BGLAP,BDKRB2,CAMK2A,PPP3CA,CEL,CPE,SDHD,LMNA,MYBPC3,CAT,CTSL,CP,MPZ,CCK,CCKAR,CHGA,CNTF,COL1A1,GJA4,CRP,CREB1,CDK4,CTLA4,IL10,AVP,HNF4A,INS,GCK,IDDM2,NIDDM4,IGF2BP2,SLC30A8,AKT2,GCGR,GPD2,HMGA1,IL6,PDX1,IRS1,KCNJ11,LIPC,MTNR1B,NEUROD1,PAX4,NIDDM3,ENPP1,PPARG,CDKAL1,RETN,PTPN1,SLC2A4,ABCC8,HNF1A,HNF1B,TCF7L2,VEGFA,WFS1,IRS2,MAPK8IP1,DUPC1,EEC1,EDS8,CDKN1C,KCNQ1OT1,H19,NSD1,SELE,EPHX2,EPO,ESR1,FRTS,FAS,FABP2,FBN1,ZLS,FOXO1,ALX3,IGAD1,GIP,GIPR,GCG,GLP1R,NR3C1,SLC2A1,SLC2A2,GAD2,CFB,GYS1,AHSG,ABCG2,GC,GZMA,HP,HT,HBA1,HBA2,HBB,NCSTN,HLA-A,HLA-B,HLA-DRB1,HLA-DRA,HTT,CETP,LPL,USF1,APOA5,HYT5,HYT6,HYT1,ADD1,CYP3A5,AGTR1,ECE1,GNB3,HYT3,HYT4,ATP1B1,NOS2,NOS3,HYT2,PTGIS,RGS5,LIPI,HTGS,RP1,IDE,HLA-DQA1,ITPR1,ITPR3,CXCL10,IGF1R,IGF1,SOD2,ICA1,INSR,IL1RN,IL2,IL1B,IL2RA,IL1A,IL6R,IAPP,LDHB,LMNB2,PTPRC,PLCD1,ALOX5,ALOX12,AKAP10,LGV1,YTHDF2,TLR4,TNFRSF8,FOXC2,YBX1,MBL2,MC3R,MC4R,MTTP,SIX3,SLC3A2,CCL2,PTEN,BMPR1A,DYNC1H1,MOG,DMPK,NRCLP,HCRT,LINC00163,IL12B,UMOD,PCSK1,MSH2,NF1,NPY,NFKB1,LEP,HFM,OAS1,RHOA,OXT,REG1A,SPINK1,CFTR,CTRC,PRSS1,PRSS2,PON1,TAP1,PLIN1,CTSC,PRPH,PPARA,PIK3R1,SERPINF1,SERPINE1,ITGB3,CD36,SELP,PARP1,POLD1,NDN,SNRPN,DLK1,PBX1,KISS1R,MNX1,HPC6,CHEK2,KLF6,HIP1,HPCQTL19,AR,CD82,HPC3,HPC4,MSR1,MXI1,ZFHX3,ELAC2,HPC5,BRCA2,PCAP,TUSC3,MAD1L1,HPCX,CDH1,POMC,MAP2K1,PTPN2,AKT1,F2,PSMB9,PSMB8,BCHE,RRAD,RBP4,PADI4,PTPN22,CIITA,NFKBIL1,CD244,SLC22A4,STAT4,RUNX1,SHBG,HESX1,ANK1,CACNA1A,FST,PCOS1,SREBF1,CD3E,CD8A,CCL5,DGCR,TBX1,CD1D,CEBPB,E2F1,ERBB3,TGFB1,TNF,TP53,TH,BLK,UBB,UGT1A1,WBSCR22,ELN,GTF2I,GTF2IRD1,XBP1,MC2R,ALMS1,APOC2,SLC2A10,VPS33B,NPHP3,ATM,AUTS1,GLO1,NRXN1,TTC8,BBS5,BBS12,TRIM32,BBS9,WDPCP,MKS1,BBS7,BBS1,BBS2,BBS4,CCDC28B,BBS10,CEP290,MKKS,ARL6,TMEM67,PCNT,BLM,NT5E,HLA-DQB1,KRT8,KRT18,C2,HRAS,KRAS,CTNS,SH2B3,FOXP3,EIF2AK3,FANCA,FANCC,FANCD2,FANCE,FANCB,FANCF,FANCG,FXN,FRDA2,KHK,GALK1,PFKM,GLYS1,SLC5A2,HFE,BMP2,HPE6,HPE1,CPS1,ABCC9,AIRE,DCAF17,B2M,UBR1,OXCT1,LMF1,SLC19A2,MUT,TRIM37,MPO,SEPN1,ACTA1,TPM3,PLA2G6,MMP2,PALLD,STK11,MANF,GH1,GHR,IL23R,DEFB4A,IBD8,IBD3,IBD9,IBD2,IBD4,IBD5,IBD6,IBD7,NOD2,DLG5,NPHP1,SETBP1,BSCL2,ZFYVE26,THM,GRD1,GRDX,GRD2,IFIH1,WRN,ARHGAP4,IDDMX,AIFM1,TRAPPC2,OGT,MECP2,MRXSL,BTK,SLC6A14,CD40LG,GK,AVPR2,CXCR3,GLA,PRPS1,ALAS2,APOO,MIR503,DKC1,NDP,TFE3,GATA6,ABCA1,PBCA,ITPR2,VANGL1,CDKN2A,GEM,MICA,AQP3,ASIP,AGER,GPR1,CCR7,AVPR1B,SLC11A1,FOXA2,AQP4,IDDM3,IDDM4,SUMO4,IDDM7,ISL1,PPARD,E2F2,CDKN2B,FABP4,TFAM,FGF8,NFATC1,P2RY12,SHC1,CASP3,EGI,EIG2,ME2,EIG3,CACNB4,HMGA2,HSD11B1,SHH,SCNN1G,TSPAN8,CDKN1B,ABCB4,AVPR1A,INPPL1,GCKR,RAB7A,IDDM8,PPP1R3A,UCN,IL18,LEPR,SCARB1,CAV1,CAV2,HK2,CYP2C9,MAPK8,TUB,IDDM11,CAV3,CLTCL1,CAPN10,NIDDM1,PPP3R1,MLRG,CYP4A11,IDDM13,BICC1,RFX6,F5,ALOX5AP,PRKCH,CCR5,VEGFB,NIDDM2,ZFP57,PLAGL1,HYMAI,TNDM,CX3CR1,SQSTM1,EZH2,TFAP2B,SMARCB1,ART1,PPARGC1B,AGRP,OB10Q,OB4,GHRL,BMIQ8,PYY,SIM1,UCP1,UCP2,UCP3,FTO,NR0B2,CARTPT,SDC3,IDDM15,PTPRN2,FKBP10,ROCK1,VDR,PTPRN,MSTN,PPP1R2,MAPK3,DGKD,LIG4,CLOCK,THRSP,IDDM6,IDDM10,CLCNKA,FUT7,PDE3B,TRPV1,KCNJ15,MYD88,DDOST,TULP2,SEL1L,HFE2,HAMP,PON2,COL6A1,OPLL,ARNTL,P2RX7,SLC22A1,TGIF1,GPR35,CNBP,CCL21,SLC29A3,MAPK9,PIK3CB,AQP7,CFHR3,CFHR1,PLEKHA1,TLR3,HMCN1,PTRF,IKBKB,IDDM17,KLF11,NDUFB6,HIF1A,PEA15,LRP5,LRP6,TNFSF10,CFLAR,APOL1,KCNE2,GFPT2,EIF2S1,DIANPH,SCD,MGEA5,PTTG1,SFRP5,ONECUT1,SIGLEC5,TFR2,SGCB,TP63,WDR62,ST3GAL5,PSTPIP1,HHEX,CIDEA,SIRT1,TFG,HMSNO,CBLB,PPARGC1A,LARS2,MGST3,NKX2-2,MGAT4A,ACTN4,RRM2B,STK17B,SOX13,ANGPTL3,NEUROG3,CISD2,GSK3B,IRF7,SOCS2,SLC38A2,HYALP,TOR1A,PDPK1,SORBS1,RNPEPL1,PFKFB3,GAD1,PRKD1,ADIPOQ,LPIN1,ATF6,FSTL1,AOMS1,PRDM16,IDDM18,MLXIPL,GPR183,ACSS2,ANGPTL4,FSI,SLC40A1,C10orf2,FADS1,SORCS1,WNT5B,NLRP3,ELMO1,GFM2,DLL1,BMIQ1,BMIQ2,BMIQ3,BMIQ4,BMIQ5,BMIQ6,HAVCR2,GP1BA,PKHD1,NAT8,CAQ5,CAQ14,MIOX,GSK3A,PAOD1,ACADS,RBM17,LDLR,IP6K1,ACADM,HDLC2,PRSS16,PTF1A,PGR,SALL4,LEPROTL1,CHDS1,MAPK8IP2,KIRREL2,DEL1P36,TRIB3,VIMP,ADIPOR1,ADIPOR2,ECI2,G6PC2,GIMAP5,MLH3,MSH6,PTGES2,AIDM1,IRGM,CHDS4,MEF2A,CYB5R4,AIS2,AIS3,STAB1,STAB2,AGPAT2,ZMPSTE24,HSPB1,INSIG2,IL33,KALRN,RPS6KB1,ADA,CRTC2,SIK2,STQTL5,ARCC1,PNPLA2,LGMD1G,IER3IP1,RC3H1,FGF21,TAGAP,SIAE,ECE2,GLIS3,NOTCH2,MAFA,PPP1R3B,ANKRD23,HDLCQ6,STRADA,CHDS8,MIR375,CLEC16A,SGIP1,FBP1,CELIAC6,NEUROD4,CLMP,THADA,AAA3,ANIB6,MRPS31,CYCS,CELIAC12,CELIAC13,FGQTL1,CIDEC,STQTL10,ZBTB38,STQTL11,CDK6,STQTL12,STQTL13,LCORL,PROS1,BMIQ14,TBC1D4,CRTAM,IDDM23,RESP18,IDDM24,CLEC7A,CTSA,CDKN2B-AS1,MIR103A1,MIR107,FGQTL2,FGQTL3,FLD1,DDX42,ITCH,VIPAS39,BMIQ16,DEL14Q11Q22,FGQTL6,LEPROT,FGQTL4,SPTLC2,SARS2,SPINT3,BANF1,GPCPD1,COL9A1,PCK1,SLC16A9,TET2,ACSS1,AAA4,C4B,C4A,COL4A1,COL4A2,DUP17Q12,DEL17Q12,CDK19,POC1A,BWQTL4,USMG5 |
| BC | FGFR2,TWIST1,PML,ZFHX3,CD44,CYP19A1,HSD17B1,CASC11,TACC3,FGFR3,HRAS,KRAS,RB1,MS4A1,BMP2,BRCA1,TFF1,BCPR,MYC,CDH3,CHEK2,AKT1,HMMR,ATM,NQO2,SLC22A18,PHB,PIK3CA,BARD1,RAD51,BRCA2,TP53,TSG101,XRCC3,PALB2,BRIP1,CASP8,RAD54L,PPM1D,RB1CC1,CDH1,CTNNB1,DCC,FLCN,EP300,MLH3,APC,SMAD7,NRAS,ODC1,PDGFRL,PLA2G2A,PTPRJ,CCND1,AURKA,BUB1B,TLR2,TLR4,AXIN2,BCL10,MET,AXIN1,CNR1,CBR1,CTNNA1,CTSL,CEBPA,CDKN1A,CDK1,CDC25A,ABCC11,MMP1,MMP2,MSH2,MLH1,CBFB,CRYAB,CCNE1,CYP2D6,CD55,NQO1,CYP24A1,DNMT1,UTRN,EGR1,GJB6,EDN1,EGFR,ESR1,FABP3,GSTP1,FGFR4,FH,GAL,KLF6,ERBB2,IL1B,IL1RN,IRF1,MUTYH,CASP10,GPT,GLS,GSTM1,SHMT2,GNRHR,GRN,CSF2,NR4A1,RASA1,CGNL1,GNAO1,HSR,HPR,HSPA2,HSP90AA1,HGF,HOXA5,HOXB9,HOXC6,HOXD10,KRT1,KRT9,KRT16,RNF139,OGG1,HNF1A,HNF1B,VHL,DIRC2,ITIH2,ITIH3,IGFBP2,IGFBP4,IGFBP5,ITIH1,IGF1R,IDO1,IGF1,IGF2,IRS1,ITGB4,JAK2,WNT2,FGFR1,TOC,RHBDF2,LOC619540,AAGAB,RUNX1,CDKN2A,ALOX12,LHCGR,FOXC2,LAG3,LSP1,LOX,PTEN,LGALS3,SERPINB5,DMBT1,ABR,SUFU,PTCH1,PTCH2,CMM,MC1R,CMM4,CXCL1,NME1,NME2,MTR,MAPT,CCL2,MUC1,BMPR1A,KMT2A,MLLT4,PTN,CXCR4,NFKB1,ETS1,ETS2,BRAF,CTTN,BMI1,REL,FGF3,WNT5A,OPCML,PARK2,ST8,PAX2,PPY,PTHLH,ADH1C,DBH,PARK10,DRD4,FGF20,GBA,NDUFV2,NR4A2,PARK3,PARK12,TBP,UCHL1,SNCAIP,ABCB1,RET,JUP,PARP1,GNAS,SMAD4,STK11,KISS1R,PIP,PRLR,HPC6,HIP1,HPCQTL19,AR,CD82,HPC3,HPC4,MSR1,MXI1,ELAC2,HPC5,PCAP,TUSC3,MAD1L1,HPCX,PTPN3,PTPN6,PTPN1,PTPRG,EPHA2,RAB6A,PTPRF,RPA1,ARID4A,RBL2,RRM1,RPN2,SPARC,SHBG,TFF2,SOX4,MMP3,CLU,SDHB,CCL5,TERT,TNC,F2R,TIMP2,PRKAR1A,CHRNA4,CYP2A6,SLC6A3,GABBR2,TFDP1,CEBPB,TGFB1,TPM1,TNFAIP3,EFNA1,UGT1A1,VCAM1,GPC3,WT1,RASSF1,MAP3K8,ERCC6,FASLG,PPP2R1B,DLEC1,FANCA,FANCC,FANCD2,FANCE,FANCB,FANCF,FANCG,PALLD,MANF,B3GALTL,SRD5A2,KIT,MGCT,DPYD,EFNB1,TEX28,IL13RA2,CTAG1B,GPC4,TGCT1,FOXP3,CTAG2,BCAP31,LDOC1,XAGE2,INGX,MAGEC2,FGD1,MST4,MIR221,MIR222,LUZP4,AMER1,FAAH2,PNCK,STARD8,BEX1,BEX2,PRAF2,MIR502,CDR1,YY1,MATK,BAX,BRCATA,TNS1,PTPN12,SYK,LCN2,DUSP3,RUNX2,FASN,CCR7,PMS2,PTGS2,PTPN13,BTC,PCGF2,ID1,XRCC2,FEN1,DDR1,AMPH,PRKCQ,AKR1C2,TRIM25,FGF8,NFE2L2,EIF4G1,ITIH4,LGALS3BP,TFF3,CASP3,SLC16A1,HMGA2,HMGA1,MPP2,TGFBR3,TFAP4,TBX2,CDKN1B,MRE11A,ST7,CXCL12,ARHGEF5,SIM2,MAP3K1,CTNND1,CAV1,FHIT,PRDM2,SIX1,MTOR,BIN1,CCR2,SHFM1,YWHAZ,SFN,MAP2K4,CSE1L,CDH13,SKP2,MAD2L1,PPARG,GRB7,GRB14,ING1,TFAP2B,TFAP2C,SMARCB1,NTN1,ESR2,ERCC2,ERCC3,GTF2H5,CYP1B1,H2AFX,MGAT5,GPER1,LIG4,SLC5A5,FAS,CST6,NEO1,SUMO1,TIMP4,THRSP,NCOA3,PTK6,EREG,NRP1,SATB1,NOVA1,SIPA1,SIAH1,ZMYM2,PER1,MMP17,FOXA1,SEL1L,BLMH,NR1H3,ROBO1,FADD,TRAF4,PAK1,MCM4,TNFSF11,TSPAN4,KLK6,MNAT1,NBN,KLK10,FOXO3,FSCN1,SDHD,NCOA1,NFATC4,RAD51C,SPG7,IGFBP7,SAFB,RBBP9,LASP1,VWA5A,BCAR1,RAD51B,RAD51D,SGK1,ZNF217,BCAS1,SNCG,ARID1A,PLAGL1,BAP1,S100A11,LMO4,CUL4A,PTPN14,SLC22A18AS,SMARCA4,HLTF,IKBKB,KISS1,BIRC5,CDKN2C,IQGAP1,WISP2,TRIM24,PER2,PEA15,DAB1,CDK10,WNT4,TNFRSF11A,MTA1,TNFSF4,TNFSF10,UNC5C,FOXH1,RPS16,CLDN1,ABCG2,DACH1,PDZK1,CBFA2T3,BAG4,FMO5,RFX4,PNMA1,RAD50,TPD52,RBBP8,IL24,ESPL1,PTTG1,SFRP1,CTCF,SOCS3,CXCL14,SNAI1,DLC1,RAD54B,MED1,MSH6,BECN1,TRPS1,SCGB2A1,KLK7,SIRT1,CBLB,LIMD1,PEBP1,HOXB13,VAT1,AKAP13,BCAR3,MERTK,HPSE,SRP68,BRAP,SLC9A3R1,FXYD3,CYLD,IKBKE,TERF2IP,MVP,RARRES3,SPO11,SPINT1,PSME3,WWOX,PXDN,PATZ1,ELF5,GAS8,C16orf3,DIRAS3,PDPK1,WNK1,NOL3,CNC,ABCC4,NCOA6,TACC1,TACC2,APPBP2,PDS5B,BRCA3,MIRLET7A1,KDM5B,CD274,ADAMTS9,ZNF350,ASPM,ITGB3BP,KLK9,KLK13,SMURF2,NRG3,HMG20B,KLK12,SCGB2A2,SERPINI2,KLK5,BACE2,PBOV1,CPNE7,HID1,COX7A2L,EBAG9,BCAS2,PES1,BBC3,ANGPTL4,PCNT,DSE,NOD1,STRAP,KEAP1,MBOAT7,NDOR1,TES,BHLHE41,GAB2,AGO2,DICER1,BRMS1,NUDT6,FBXW7,AGR2,GLS2,HTRA2,CSRNP1,UGT2B28,SCGB3A1,CASC3,CYP27A1,LZTS1,SLC36A1,FRK,DCD,NLRP1,CEACAM19,PKHD1,TTC4,ST13,ST14,IL17RD,CITED4,TNS3,PYCARD,HIPK2,PHGDH,RBM5,TNK2,LPHN2,CTCFL,ABCC12,CLN3,STARD3,LRRK2,ZMYND10,CACNA2D2,WHSC1L1,MTHFR,APOBEC3B,TNKS2,CREB3L4,MN1,NF2,SEDM8,LSM1,PGR,LOXL4,RHOBTB2,CCAR2,WWTR1,EPSTI1,PPP1R13L,BCAS3,BCAS4,PIBF1,DIS3,BPIFA4P,RNF20,C8orf4,TJP2,DDIT4,SESN2,UNC5A,UNC5B,OVCA2,KCNRG,PRSS50,RANGRF,SASH1,OSGIN1,S100A14,UHRF1,ASH1L,HIPK1,GNL3,HSPB8,TSKU,COA4,FAM162A,SAFB2,ERRFI1,CENPV,PAXIP1,IGF2BP1,CAGE1,EMCN,LIMA1,TNS4,UBR5,MTSS1,CYBA,RSF1,ING4,C11orf30,PPIAL4E,PDCD4,PSMC3IP,PTPRT,SLC39A6,BRD4,POSTN,SMYD3,BLID,RND1,MTA3,CARD8,FBXO31,FBXO45,CLDN7,AKIP1,DAB2IP,CYP17A1,MIR155,ARL11,PHLPP1,ARHGAP8,MIR17,NDUFA13,PELP1,AGR3,FAM84B,LYPD3,CYP27B1,KLF17,PVRL4,MDGA1,DDX58,TARP,ARID4B,MIR16-1,PDLIM2,NCOA7,KDM4B,ITIH5,NEK8,STARD13,CASZ1,CDCA7,VPS45,SARNP,CHMP3,RINT1,MIR125B1,MIR125B2,ST6GALNAC5,ST6GALNAC2,ST6GALNAC1,ST8SIA6,TBC1D3B,KIAA1009,CRIPAK,SLIRP,ORAI1,GALNT12,MED28,MDMH,SLC2A12,ACKR3,MACROD1,NTN4,LINC00312,TTC9,WWC1,RSPO3,MIR27B,KIAA0100,PHB2,PDRG1,SLC25A33,FAM102A,HPC9,MEX3C,MEX3A,MEX3B,TMEM259,MIR21,PPFIA1,MIR34A,PBK,UNC45A,AKT3,BNIPL,KIF14,PRICKLE4,HOTAIR,TOX3,CIZ1,C3orf35,BANP,MIR10B,DDX54,ADAMTS20,GREB1,SCUBE2,MIR126,MIR335,NDUFAF4,PRDM14,ABCB5,PGAP3,MIEN1,BCCIP,S100PBP,C14orf169,ATAD2,MIR373,MIR200A,MIR200B,MIR200C,MIR141,MIR429,KLLN,TNFAIP8,RASL10B,GLCE,ECHDC1,PREX2,GOLPH3,MRC2,ARRDC3,PEBP4,RNF31,CCAR1,RNF11,RASSF6,TCHP,RERG,PYHIN1,UTP20,NOP16,RAB6C,TMEM97,RAB25,ATP2C2,MIR205,SUV420H2,TAOK2,PDS5A,KCDM3,SAE1,UBA2,LAPTM4B,ARHGAP18,DDX42,TMEM127,CCDC62,SPEN,SXGQTL1,LRRC26,ATG14,KIAA0226,CLEC3A,MIR661,BCAR4,SLC36A4,SCAMP5,UQCRH,TOE1,ARGLU1,RHNO1,MCIDAS,PAPD4,CUEDC2,CHTOP,SLC16A9,DMRD3,DHX33,AFAP1L1,MIR1258,KLHDC8A,BRAT1,ATR,ZDHHC7,ZDHHC21,AMER2,TSPYL5,MIR193B,ODAM,MIR298,ATPIF1,EPG5,LY6K,MIR495,MIR30C1,RNF126,RHBDD2,MIR7-1 |
| T2DM | STAT3,DPP4,GNAS,APP,AGT,ACE,PAX6,APOA1,APOE,CYP19A1,ARRB2,ATXN3,ADRB2,ABO,BGLAP,BDKRB2,CAMK2A,PPP3CA,CEL,SDHD,LMNA,MYBPC3,CAT,CTSL,MPZ,CHGA,COL1A1,CREB1,CTLA4,IL10,AVP,AQP2,HNF4A,INS,IDDM2,NIDDM4,IGF2BP2,SLC30A8,AKT2,GCGR,GCK,GPD2,HMGA1,IL6,PDX1,IRS1,KCNJ11,LIPC,MTNR1B,NEUROD1,PAX4,NIDDM3,ENPP1,PPARG,CDKAL1,RETN,PTPN1,SLC2A4,ABCC8,HNF1A,HNF1B,TCF7L2,VEGFA,WFS1,IRS2,MAPK8IP1,EDS8,CDKN1C,KCNQ1OT1,H19,NSD1,SELE,EPHX2,EPO,ESR1,FRTS,FAS,FABP2,FBN1,IGAD1,NR3C1,SLC2A1,SLC2A2,GAD2,CFB,HBA1,HBA2,HBB,HLA-A,HLA-B,HLA-DRB1,HLA-DRA,HTT,CETP,LPL,USF1,HYT5,HYT6,HYT1,ADD1,CYP3A5,AGTR1,ECE1,GNB3,HYT3,HYT4,ATP1B1,NOS2,NOS3,HYT2,PTGIS,RGS5,APOA5,LIPI,HTGS,RP1,IDE,HLA-DQA1,IGF1R,IGF1,SOD2,INSR,IL1A,IAPP,LDHB,PTPRC,FOXC2,YBX1,MC4R,MTTP,SLC3A2,CCL2,DYNC1H1,DMPK,NRCLP,HCRT,LINC00163,MSH2,NF1,NPY,NFKB1,LEP,HFM,RHOA,REG1A,SPINK1,TAP1,CTSC,PRPH,PPARA,SERPINE1,CD36,SELP,PARP1,NDN,SNRPN,F2,PSMB9,PSMB8,BCHE,RRAD,PADI4,PTPN22,CIITA,NFKBIL1,CD244,SLC22A4,STAT4,RUNX1,FST,PCOS1,CD1D,CEBPB,ERBB3,TGFB1,TNF,TP53,TH,UBB,UGT1A1,WBSCR22,ELN,GTF2I,GTF2IRD1,XBP1,ALMS1,APOC2,NPHP3,ATM,AUTS1,GLO1,NRXN1,TTC8,BBS5,BBS12,TRIM32,BBS9,WDPCP,MKS1,BBS7,BBS1,BBS2,BBS4,CCDC28B,BBS10,CEP290,MKKS,ARL6,TMEM67,PCNT,BLM,HLA-DQB1,C2,HRAS,KRAS,SH2B3,ITPR3,OAS1,FOXP3,FANCA,FANCC,FANCD2,FANCE,FANCB,FANCF,FANCG,BRCA2,FXN,FRDA2,PFKM,HFE,BMP2,ABCC9,AIRE,LMF1,MUT,PALLD,STK11,MANF,GH1,GHR,IL23R,DEFB4A,IBD8,IBD3,IBD9,IBD2,IBD4,IBD5,IBD6,IBD7,NOD2,DLG5,GRD1,GRDX,GC,GRD2,IFIH1,AR,AIFM1,OGT,BTK,SLC6A14,CD40LG,AVPR2,ABCA1,CDKN2A,GEM,ASIP,AGER,ISL1,PPARD,SHC1,HMGA2,HSD11B1,SHH,CDKN1B,GCKR,RAB7A,PPP1R3A,LEPR,SCARB1,CAV1,HK2,CYP2C9,CAPN10,NIDDM1,NIDDM2,ZFP57,PLAGL1,HYMAI,TNDM,PPARGC1B,ADRB3,AGRP,OB10Q,OB4,MC3R,GHRL,POMC,BMIQ8,PYY,SIM1,UCP1,UCP2,UCP3,FTO,NR0B2,CARTPT,SDC3,VDR,MSTN,PPP1R2,LIG4,CLOCK,FUT7,DDOST,SEL1L,CFTR,ARNTL,MAPK9,CFHR3,CX3CR1,CFHR1,PLEKHA1,TLR3,HMCN1,IKBKB,KLF11,HIF1A,PEA15,LRP5,LRP6,TNFSF10,GFPT2,EIF2S1,DIANPH,ONECUT1,ST3GAL5,PPARGC1A,MGST3,WRN,MGAT4A,ANGPTL3,SOCS2,SORBS1,ADIPOQ,ATF6,FSTL1,AOMS1,FSI,NLRP3,DLL1,BMIQ1,BMIQ2,BMIQ3,BMIQ4,BMIQ5,BMIQ6,GP1BA,GSK3A,LDLR,IP6K1,HDLC2,CHDS1,DEL1P36,VIMP,MLH3,MSH6,PTEN,CDH1,HFE2,AGPAT2,IL33,KALRN,RPS6KB1,LGMD1G,ECE2,PPP1R3B,CLMP,PROS1,BMIQ14,APOL1,SARS2,GPCPD1,PCK1 |

**Supplemental Table 2**

**2.1:Overlapping module 9 breast cancer and diabetes**(P<0.05and top10)

| **Module** | **GOTERM_BP_FAT** | | **KEGG_PATHWAY** | |
| --- | --- | --- | --- | --- |
|  | **Term** | **P-Value** | **Term** | **P-Value** |
| M_b11.d12.2d11_ | GO:0006486~protein amino acid glycosylation | 1.6E-5 | hsa00601:Glycosphingolipid biosynthesis | 2.4E-2 |
|  | GO:0070085~glycosylation | 1.6E-5 |  |  |
|  | GO:0043413~biopolymer glycosylation | 1.6E-5 |  |  |
|  | GO:0009101~glycoprotein biosynthetic process | 3.0E-5 |  |  |
|  | GO:0009100~glycoprotein metabolic process | 6.3E-5 |  |  |
|  | GO:0033692~cellularpolysaccharidebiosynthetic | 1.1E-2 |  |  |
|  | GO:0006487~protein amino acid N-linked glycosylation | 1.9E-2 |  |  |
|  | GO:0000271~polysaccharide biosynthetic process | 2.0E-2 |  |  |
|  | GO:0044264~cellular polysaccharide metabolic process | 2.3E-2 |  |  |
|  | GO:0034637~cellular carbohydrate biosynthetic process | 3.0E-2 |  |  |
| M_b26.d32_ | GO:0006120~mitochondrial electron transport, NADH to ubiquinone | 2.8E-8 | hsa05012:Parkinson's disease | 1.6E-5 |
|  | GO:0042775~mitochondrial ATP synthesis coupled electron transport | 6.7E-8 |  |  |
|  | GO:0042773~ATP synthesis coupled electron transport | 6.7E-8 | hsa00190:Oxidative phosphorylation | 1.6E-5 |
|  | GO:0022904~respiratory electron transport chain | 1.0E-7 |  |  |
|  | GO:0045333~cellular respiration | 3.6E-7 |  |  |
|  | GO:0006119~oxidative phosphorylation | 3.7E-7 | hsa05010:Alzheimer's disease | 3.2E-5 |
|  | GO:0022900~electron transport chain | 5.8E-7 |  |  |
|  | GO:0015980~energy derivation by oxidation of organic compounds | 1.2E-6 |  |  |
|  | GO:0006091~generation of precursor metabolites and energy | 1.2E-5 | hsa05016:Huntington's disease | 4.4E-5 |
|  | GO:0055114~oxidation reduction | 1.0E-4 |  |  |
| M_b52.d50_ | - |  | - |  |
| M_b70.d67_ | GO:0009719~response to endogenous stimulus | 2.6E-3 | hsa04730:Long-term depression | 1.4E-2 |
|  | GO:0007212~dopamine receptor signaling pathway | 4.0E-3 |  |  |
|  | GO:0043547~positive regulation of GTPase activity | 4.9E-3 |  |  |
|  | GO:0010033~response to organic substance | 8.2E-3 |  |  |
|  | GO:0043087~regulation of GTPase activity | 2.7E-2 |  |  |
|  | GO:0030900~forebrain development | 3.3E-2 |  |  |
|  | GO:0051345~positive regulation of hydrolase activity | 3.9E-2 |  |  |
|  | GO:0010035~response to inorganic substance | 4.5E-2 |  |  |
|  | GO:0042493~response to drug | 4.7E-2 |  |  |
| M_b105.d89.2d68_ | GO:0006914~autophagy | 5.6E-3 | hsa04142:Lysosome | 5.2E-4 |
| M_b136.d107_ | - |  | - |  |
| M_b153.d141.2d99_ | - |  | - |  |
| M_b157.d147_ | - |  | - |  |
| M_b159.d146_ | - |  | - |  |

2.2:**Overlapping module 9 breast cancer and type II diabetes**(P<0.05and top10)

| **Module** | **GOTERM_BP_FAT** | | **KEGG_PATHWAY** | |
| --- | --- | --- | --- | --- |
|  | **Term** | **P-Value** | **Term** | **P-Value** |
| M_b8.2d8_ | GO:0008202~steroid metabolic process | 3.3E-6 | hsa00053:Ascorbate and aldarate metabolism | 8.6E-11 |
|  | GO:0006805~xenobiotic metabolic process | 6.2E-6 | hsa00040:Pentose and glucuronate  interconversions | 1.1E-10 |
|  | GO:0009410~response to xenobiotic stimulus | 8.3E-6 | hsa00860:Porphyrin and chlorophyll metabolism | 1.5E-9 |
|  | GO:0008210~estrogen metabolic process | 2.2E-3 | hsa00150:Androgen and estrogen metabolism | 2.4E-9 |
|  | GO:0034754~cellular hormone metabolic process | 1.3E-2 | hsa00500:Starch and sucrose metabolism | 4.0E-9 |
|  | GO:0042445~hormone metabolic process | 2.3E-2 | hsa00983:Drug metabolism | 4.4E-9 |
|  | GO:0010817~regulation of hormone levels | 3.3E-2 | hsa00140:Steroid hormone biosynthesis | 5.9E-9 |
|  |  |  | hsa00830:Retinol metabolism | 1.1E-8 |
|  |  |  | hsa00980:Metabolism of xenobiotics by cytochrome P450 | 1.8E-8 |
|  |  |  | hsa00982:Drug metabolism | 2.0E-8 |
| M_b11.d12.2d11_ | GO:0006486~protein amino acid glycosylation | 1.6E-5 | hsa00601:Glycosphingolipid biosynthesis | 2.4E-2 |
|  | GO:0070085~glycosylation | 1.6E-5 |  |  |
|  | GO:0043413~biopolymer glycosylation | 1.6E-5 |  |  |
|  | GO:0009101~glycoprotein biosynthetic process | 3.0E-5 |  |  |
|  | GO:0009100~glycoprotein metabolic process | 6.3E-5 |  |  |
|  | GO:0033692~cellularpolysaccharidebiosynthetic process | 1.1E-2 |  |  |
|  | GO:0006487~protein amino acid N-linked glycosylation | 1.9E-2 |  |  |
|  | GO:0000271~polysaccharide biosynthetic process | 2.0E-2 |  |  |
|  | GO:0044264~cellular polysaccharide metabolic process | 2.3E-2 |  |  |
|  | GO:0034637~cellular carbohydrate biosynthetic process | 3.0E-2 |  |  |
| M_b105.d89.2d68_ | GO:0006914~autophagy | 5.6E-3 | hsa04142:Lysosome | 5.2E-4 |
| M_b139.2d78_ | - |  | - |  |
| M_b153.d141.2d99_ | - |  | - |  |

**2.3:Diabetes and type Ⅱ diabetes 40 overlap module of the top five(P<0.05 and top 10)**

| **Module** | **GOTERM_BP_FAT** | | **KEGG_PATHWAY** | |
| --- | --- | --- | --- | --- |
|  | **Term** | **P-Value** | **Term** | **P-Value** |
| M_2d5.d5_ | GO:0008038~neuron recognition | 1.1E-2 | - |  |
|  | GO:0019226~transmission of nerve impulse | 1.3E-2 |  |  |
|  | GO:0050877~neurological system process | 1.9E-2 |  |  |
|  | GO:0008037~cell recognition | 2.8E-2 |  |  |
| M_2d16.d21_ | GO:0006812~cation transport | 6.4E-4 | - |  |
|  | GO:0006811~ion transport | 1.7E-3 |  |  |
|  | GO:0030001~metal ion transport | 1.1E-2 |  |  |
|  | GO:0055085~transmembrane transport | 1.6E-2 |  |  |
|  | GO:0000041~transition metal ion transport | 2.6E-2 |  |  |
| M_2d17.d20_ | GO:0007049~cell cycle | 5.1E-5 | hsa04110:Cell cycle | 5.7E-3 |
|  | GO:0006260~DNA replication | 1.9E-3 |  |  |
|  | GO:0051301~cell division | 4.5E-3 |  |  |
|  | GO:0000278~mitotic cell cycle | 7.1E-3 |  |  |
|  | GO:0022403~cell cycle phase | 8.8E-3 |  |  |
|  | GO:0006259~DNA metabolic process | 1.3E-2 | hsa03030:DNA replication | 3.5E-2 |
|  | GO:0022402~cell cycle process | 1.6E-2 |  |  |
|  | GO:0000079~regulation of cyclin-dependent protein kinase activity | 2.0E-2 |  |  |
|  | GO:0006261~DNA-dependent DNA replication | 2.1E-2 |  |  |
|  | GO:0051329~interphase of mitotic cell cycle | 3.7E-2 |  |  |
| M_2d20.d17_ | GO:0008366~axon ensheathment | 9.4E-5 | - |  |
|  | GO:0007272~ensheathment of neurons | 9.4E-5 |  |  |
|  | GO:0010001~glial cell differentiation | 1.5E-4 |  |  |
|  | GO:0019228~regulation of action potential in neuron | 1.6E-4 |  |  |
|  | GO:0045944~positive regulation of transcription from RNA polymerase II promoter | 2.0E-4 |  |  |
|  | GO:0042063~gliogenesis | 2.3E-4 |  |  |
|  | GO:0001508~regulation of action potential | 2.5E-4 |  |  |
|  | GO:0045893~positive regulation of transcription, DNA-dependent | 4.1E-4 |  |  |
|  | GO:0051254~positive regulation of RNA metabolic process | 4.2E-4 |  |  |
|  | GO:0045941~positive regulation of transcription | 6.8E-4 |  |  |
| M_b11.d12.2d11_ | GO:0006486~protein amino acid glycosylation | 1.6E-5 | hsa00601:Glycosphingolipid biosynthesis | 2.4E-2 |
|  | GO:0070085~glycosylation | 1.6E-5 |  |  |
|  | GO:0043413~biopolymer glycosylation | 1.6E-5 |  |  |
|  | GO:0009101~glycoprotein biosynthetic process | 3.0E-5 |  |  |
|  | GO:0009100~glycoprotein metabolic process | 6.3E-5 |  |  |
|  | GO:0033692~cellularpolysaccharidebiosynthetic process | 1.1E-2 |  |  |
|  | GO:0006487~protein amino acid N-linked glycosylation | 1.9E-2 |  |  |
|  | GO:0000271~polysaccharide biosynthetic process | 2.0E-2 |  |  |
|  | GO:0044264~cellular polysaccharide metabolic process | 2.3E-2 |  |  |
|  | GO:0034637~cellular carbohydrate biosynthetic process | 3.0E-2 |  |  |

**2.4:The BC/TDM/T2DM non-overlapping find seven modules in the first 10 points more than seven modules(P<0.05and top10)**

| **Module** | **GOTERM_BP_FAT** | | | **KEGG_PATHWAY** | |
| --- | --- | --- | --- | --- | --- |
|  | **Term** | | **P-Value** | **Term** | **P-Value** |
| M_b1_ | GO:0007242~intracellular signaling cascade | | 1.6E-7 | hsa04510:Focal adhesion | 7.6E-5 |
|  | GO:0007266~Rho protein signal transduction | | 2.0E-7 | hsa05211:Renal cell carcinoma | 8.4E-5 |
|  | GO:0007010~cytoskeleton organization | | 1.2E-6 | hsa05212:Pancreatic cancer | 9.1E-5 |
|  | GO:0030036~actin cytoskeleton organization | | 1.2E-5 | hsa04370:VEGF signaling pathway | 1.0E-4 |
|  | GO:0007265~Ras protein signal transduction | | 1.2E-5 | hsa04666:Fc gamma R-mediated phagocytosis | 2.1E-4 |
|  | GO:0030029~actin filament-based process | | 1.6E-5 | hsa04660:T cell receptor signaling pathway | 3.1E-4 |
|  | GO:0007264~small GTPase mediated signal transduction | | 5.1E-5 | hsa04670:Leukocyte transendothelial migration | 4.0E-4 |
|  | GO:0030334~regulation of cell migration | | 7.9E-5 | hsa04722:Neurotrophin signaling pathway | 4.6E-4 |
|  | GO:0040012~regulation of locomotion | | 1.3E-4 | hsa04360:Axon guidance | 5.2E-4 |
|  | GO:0051270~regulation of cell motion | | 1.3E-4 | hsa04062:Chemokine signaling pathway | 1.5E-3 |
| M_b2_ | GO:0006091~generation of precursor metabolites and energy | | 1.1E-16 | hsa05016:Huntington's disease | 2.1E-10 |
|  | GO:0006119~oxidative phosphorylation | | 6.2E-10 | hsa05012:Parkinson's disease | 9.0E-10 |
|  | GO:0007267~cell-cell signaling | | 5.4E-9 | hsa00190:Oxidative phosphorylation | 1.1E-9 |
|  | GO:0045333~cellular respiration | | 9.0E-9 | hsa05010:Alzheimer's disease | 2.0E-7 |
|  | GO:0015980~energy derivation by oxidation of organic compounds | | 3.9E-7 | hsa04060:Cytokine-cytokine receptor interaction | 1.2E-5 |
|  | GO:0022900~electron transport chain | | 5.2E-7 | hsa00020:Citrate cycle (TCA cycle) | 1.4E-3 |
|  | GO:0022904~respiratory electron transport chain | | 1.1E-6 | hsa00030:Pentose phosphate pathway | 7.0E-3 |
|  | GO:0042127~regulation of cell proliferation | | 2.5E-6 | hsa00010:Glycolysis / Gluconeogenesis | 1.5E-2 |
|  | GO:0042773~ATP synthesis coupled electron transport | | 7.6E-6 | hsa04260:Cardiac muscle contraction | 3.5E-2 |
|  | GO:0042775~mitochondrial ATP synthesis coupled electron transport | | 7.6E-6 | hsa04672:Intestinal immune network for IgA production | 4.2E-2 |
| M_b3_ | GO:0042127~regulation of cell proliferation | | 7.6E-14 | hsa02010:ABC transporters | 1.6E-12 |
|  | GO:0008285~negative regulation | | 8.3E-11 | hsa05210:Colorectal cancer | 1.7E-10 |
|  | GO:0008637~apoptotic mitochondrial changes | | 4.3E-9 | hsa05200:Pathways in cancer | 4.7E-6 |
|  | GO:0001836~release of cytochrome c from mitochondria | | 1.2E-8 | hsa05220:Chronic myeloid leukemia | 2.5E-4 |
|  | GO:0008219~cell death | | 6.0E-8 | hsa05213:Endometrial cancer | 3.5E-4 |
|  | GO:0010033~response to organic substance | | 6.3E-8 | hsa04115:p53 signaling pathway | 1.2E-3 |
|  | GO:0016265~death | | 6.8E-8 | hsa05219:Bladder cancer | 1.5E-3 |
|  | GO:0046649~lymphocyte activation | | 7.3E-8 | hsa04660:T cell receptor signaling pathway | 1.7E-3 |
|  | GO:0001775~cell activation | | 8.1E-8 | hsa04310:Wnt signaling pathway | 9.0E-3 |
|  | GO:0009719~response to endogenous stimulus | | 1.7E-7 | hsa05212:Pancreatic cancer | 1.1E-2 |
| M_d1_ | GO:0006955~immune response | | 9.9E-9 | hsa04060:Cytokine-cytokine receptor interaction | 1.3E-12 |
|  | GO:0007267~cell-cell signaling | | 1.1E-5 | hsa04672:Intestinal immune network for IgA production | 2.6E-3 |
|  | GO:0001817~regulation of cytokine production | | 1.9E-5 | hsa04512:ECM-receptor interaction | 1.2E-2 |
|  | GO:0009611~response to wounding | | 2.8E-5 | hsa05330:Allograft rejection | 1.8E-2 |
|  | GO:0006954~inflammatory response | | 5.7E-5 | hsa05332:Graft-versus-host disease | 2.0E-2 |
|  | GO:0006952~defense response | | 9.1E-5 | hsa04062:Chemokine signaling pathway | 2.0E-2 |
|  | GO:0010604~positive regulation of macromolecule metabolic process | | 2.3E-4 | hsa04510:Focal adhesion | 2.6E-2 |
|  | GO:0042102~positive regulation of T cell proliferation | | 2.4E-4 | hsa05320:Autoimmune thyroid disease | 3.4E-2 |
|  | GO:0010647~positive regulation of cell communication | | 5.0E-4 | hsa04621:NOD-like receptor signaling pathway | 4.8E-2 |
|  | GO:0050671~positive regulation of lymphocyte proliferation | | 6.5E-4 |  |  |
| M_d2_ | GO:0042127~regulation of cell proliferation | | 1.2E-8 | hsa04115:p53 signaling pathway | 3.3E-4 |
|  | GO:0045165~cell fate commitment | | 1.3E-6 | hsa00620:Pyruvate metabolism | 5.4E-3 |
|  | GO:0010604~positive regulation of macromolecule metabolic process | | 5.8E-6 | hsa04310:Wnt signaling pathway | 1.1E-2 |
|  | GO:0051960~regulation of nervous system development | | 1.4E-5 | hsa05213:Endometrial cancer | 1.1E-2 |
|  | GO:0006107~oxaloacetate metabolic process | | 2.1E-5 | hsa05217:Basal cell carcinoma | 1.3E-2 |
|  | GO:0045596~negative regulation of cell differentiation | | 3.3E-5 | hsa05214:Glioma | 1.9E-2 |
|  | GO:0048878~chemical homeostasis | | 3.4E-5 | hsa05218:Melanoma | 2.6E-2 |
|  | GO:0001503~ossification | | 5.2E-5 | hsa05200:Pathways in cancer | 2.6E-2 |
|  | GO:0048598~embryonic morphogenesis | | 6.6E-5 | hsa05216:Thyroid cancer | 2.8E-2 |
|  | GO:0060348~bone development | | 7.5E-5 | hsa05220:Chronic myeloid leukemia | 3.0E-2 |
| M_2d1_ | GO:0006955~immune response | | 9.9E-9 | hsa04060:Cytokine-cytokine receptor interaction | 1.3E-12 |
|  | GO:0007267~cell-cell signaling | | 1.1E-5 | hsa04672:Intestinal immune network for IgA production | 2.6E-3 |
|  | GO:0001817~regulation of cytokine production | | 1.9E-5 | hsa04512:ECM-receptor interaction | 1.2E-2 |
|  | GO:0009611~response to wounding | | 2.8E-5 | hsa05330:Allograft rejection | 1.8E-2 |
|  | GO:0006954~inflammatory response | | 5.7E-5 | hsa05332:Graft-versus-host disease | 2.0E-2 |
|  | GO:0006952~defense response | | 9.1E-5 | hsa04062:Chemokine signaling pathway | 2.0E-2 |
|  | GO:0010604~positive regulation of macromolecule metabolic process | | 2.3E-4 | hsa04510:Focal adhesion | 2.6E-2 |
|  | GO:0042102~positive regulation of T cell proliferation | | 2.4E-4 | hsa05320:Autoimmune thyroid disease | 3.4E-2 |
|  | GO:0010647~positive regulation of cell communication | | 5.0E-4 | hsa04621:NOD-like receptor signaling pathway | 4.8E-2 |
|  | GO:0050671~positive regulation of lymphocyte proliferation | | 6.5E-4 |  |  |
| M_2d2_ | - |  | | - |  |

**Supplemental Table 3**

**56G**enes related to disease in literature.

| **Gene of disease** | **BC** | **DM** | **T2DM** |
| --- | --- | --- | --- |
| CYP19A1 | [1].[24] | [1] | - |
| HRAS | [2] | [3] | - |
| KRAS | [4] | [5] | - |
| BMP2 | [6] | [7] | [8] |
| ATM | [9].[22] | [10] | [11] |
| BRCA2 | [19].[24].[25] | [24].[25] | - |
| TP53 | [11].[19].[22] | [12] | [13] |
| CDH1 | [14].[22] | [17] | [17] |
| MLH3 | [18].[19] | - | - |
| CTSL | [20] | [21] | - |
| MSH2 | [22] | - | [23] |
| ESR1 | [24] | [25] | [26] |
| HNF1A | [27] | [28] | [29] |
| HNF1B | [30] | [31] | [32] |
| IGF1R | [15].[16] | [33] | [34] |
| IGF1 | [35] | [36] | [37] |
| IRS1 | [38] | [39] | [39] |
| RUNX1 | [40] | [41] | [42] |
| CDKN2A | [14].[22] | [43] | [44] |
| FOXC2 | [45] | [46] | [47] |
| PTEN | [14].[22].[48] | [49] | [50] |
| CCL2 | [51] | [52] | [53] |
| NFKB1 | [54] | [55] | [56] |
| PARP1 | [57] | [58] | [59] |
| GNAS | [60] | [61] | [62] |
| STK11 | [63] | [64] | [65] |
| AR | [66] | [67] | [68] |
| PTPN1 | [69] | [70] | [71] |
| CEBPB | [72] | [73] | [74] |
| TGFB1 | [75] | [76] | [77] |
| UGT1A1 | [78] | - | - |
| FANCA | [79].[83] | [80] | - |
| FANCC | [81].[83] | - | - |
| FANCD2 | [82] | - | - |
| FANCE | [83] | - | - |
| FANCB | [84] | - |  |
| FANCF | [83] | - | [85] |
| FANCG | [83] | - | - |
| PALLD | [86] | [87] | - |
| MANF | - | - | - |
| FOXP3 | [88] | [89] | - |
| HMGA2 | [90] | - | - |
| HMGA1 | [91] | [92] | [93] |
| CDKN1B | [94] | [95] | [95] |
| CAV1 | [96] | [97] | - |
| PPARG | [98] | [99] | [100] |
| LIG4 | [101] | [102] | - |
| FAS | [103] | [104] | [105] |
| SEL1L | [106] | [107] | - |
| SDHD | [108] | [109] | - |
| PLAGL1 | [110] | [111] | [112] |
| IKBKB | [113] | [114] | [115] |
| PEA15 | [116] | [117] | [118] |
| TNFSF10 | [119] | [120] | [121] |
| MSH6 | [18].[22] | - | - |
| PCNT | - | [122] | - |

1. **Stueve TR, Wolff MS, Pajak A, *et al*.** CYP19A1 promoter methylation in saliva associated with milestones of pubertal timing in urban girls. *BMC Pediatr*. 2014;14: 78.

2. **Kai K, Iwamoto T, Kobayashi T, *et al*.** Ink4a/Arf(-/-) and HRAS(G12V) transform mouse mammary cells into triple-negative breast cancer containing tumorigenic CD49f(-) quiescent cells. *Oncogene*. 2014; 33: 440-8.

3. **Elbein SC, Corsetti L, Goldgar D, *et al*.** Insulin gene in familial NIDDM. Lack of linkage in Utah Mormon pedigrees. *Diabetes*. 1988; 37: 569-76.

4. **Lyu S, Yu Q, Ying G, *et al*.** Androgen receptor decreases CMYC and KRAS expression by upregulating let-7a expression in ER-, PR-, AR+ breast cancer. *Int J Oncol*. 2014; 44: 229-37.

5. **Fujimoto T, Shirasawa S.** KRAS-induced actin-interacting protein: a potent target for obesity, diabetes and cancer. *Anticancer Res*. 2011; 31: 2413-7.

6. **Buijs JT, van der Horst G, van den Hoogen C, *et al*.** The BMP2/7 heterodimer inhibits the human breast cancer stem cell subpopulation and bone metastases formation. *Oncogene*. 2012; 31: 2164-74.

7. **Hussein KA, Choksi K, Akeel S, *et al*.** Bone morphogenetic protein 2: A potential new player in the pathogenesis of diabetic retinopathy. *Exp Eye Res*. 2014.

8. **Al-Aly Z, Shao JS, Lai CF, *et al*.** Aortic Msx2-Wnt calcification cascade is regulated by TNF-alpha-dependent signals in diabetic Ldlr-/- mice. *Arterioscler Thromb Vasc Biol.* 2007; 27: 2589-96.

9. **Singh R, Shankar BS, Sainis KB.** TGF-β1-ROS-ATM-CREB signaling axis in macrophage mediated migration of human breast cancer MCF7 cells. *Cell Signal*. 2014; 26: 1604-15.

10. **Nagareddy PR, Kraakman M, Masters SL, *et al*.** Adipose tissue macrophages promote myelopoiesis and monocytosis in obesity. *Cell Metab*. 2014; 19: 821-35.

11. **Shin E, Shin S, Kong H, *et al*.** Dietary Aloe Reduces Adipogenesis via the Activation of AMPK and Suppresses Obesity-related Inflammation in Obese Mice. *Immune Netw*. 2011; 11: 107-13.

11. **Jiang YZ, Yu KD, Bao J, *et al*.** Favorable Prognostic Impact in Loss of TP53 and PIK3CA Mutations after Neoadjuvant Chemotherapy in Breast Cancer. *Cancer Res*. 2014.

12. **Spitsina EV, Iakunina NIu, Chudakova DA, *et al*.** Association of polymorphous markers Pro72Arg and C(-594)CC OF TP53 gene with diabetic polyneuropathy in patients with type 1 diabetes mellitus living in Moscow. *Mol Biol (Mosk).* 2007; 41: 989-93.

13. **Qu L, He B, Pan Y, *et al.*** Association between polymorphisms in RAPGEF1, TP53, NRF1 and type 2 diabetes in Chinese Han population. *Diabetes Res Clin Pract*. 2011; 91: 171-6.

14. **Wheler JJ, Parker BA, Lee JJ, *et al*.** Unique molecular signatures as a hallmark of patients with metastatic breast cancer: Implications for current treatment paradigms. *Oncotarget*. 2014; 5: 2349-54.

15. **SusanLNeuhausen, SeanBrummel, YuanChunDing, *et al*.** Genetic variation in insulin-like growth factor signaling genes and breast cancer risk among BRCA1 and BRCA2 carriers. *Breast Cancer Research*. 2009; 11: R76.

16. **Bordeleau L, Lipscombe L, Lubinski J, *et al.*** Diabetes and breast cancer among women with BRCA1 and BRCA2 mutations. *Cancer*. 2011; 117: 1812-8.

17. **Lees CW, Barrett JC, Parkes M, *et al*.** New IBD genetics: common pathways with other diseases. *Gut*. 2011; 60: 1739-53.

18. **Conde J, Silva SN, Azevedo AP, *et al.*** Association of common variants in mismatch repair genes and breast cancer susceptibility: a multigene study. *BMC Cancer*. 2009; 9: 344.

19. **Maia AT, Spiteri I, Lee AJ, *et al.*** Extent of differential allelic expression of candidate breast cancer genes is similar in blood and breast. *Breast Cancer Res*. 2009; 11: R88.

20. **Croke M, Neumann MA, Grotsky DA, *et al*.** Differences in 53BP1 and BRCA1 regulation between cycling and non-cycling cells. *Cell Cycle.* 2013; 12: 3629-39.

21. **Hsing LC, Kirk EA, McMillen TS,** ***et al*.** Roles for cathepsins S, L, and B in insulitis and diabetes in the NOD mouse. *J Autoimmun*. 2010; 34: 96-104.

22. **Silva FC, Lisboa BC, Figueiredo MC, *et al*.** Hereditary breast and ovarian cancer: assessment of point mutations and copy number variations in Brazilian patients. *BMC Med Genet*. 2014; 15: 55.

23. **Switzeny OJ, Müllner E, Wagner KH,** ***et al*.** Vitamin and antioxidant rich diet increases MLH1 promoter DNA methylation in DMT2 subjects. *Clin Epigenetics.* 2012; 4: 19.

24. **Chattopadhyay S, Siddiqui S, Akhtar MS,** ***et al*.** Genetic polymorphisms of ESR1, ESR2, CYP17A1, and CYP19A1 and the risk of breast cancer: a case control study from North India. *Tumour Biol.* 2014; 35: 4517-27.

25. **Moore K, Ghatnekar G, Gourdie RG, *et al*.** Impact of the controlled release of a connexin 43 peptide on corneal wound closure in an STZ model of type I diabetes. *PLoS One.* 2014; 9: e86570.

26. **Hale PJ, López-Yunez AM, Chen JY.** Genome-wide meta-analysis of genetic susceptible genes for Type 2 Diabetes. *BMC Syst Biol.* 2012; 6: S16.

27. **Prizment AE, Folsom AR, Dreyfus J, *et al*.** Plasma C-reactive protein, genetic risk score, and risk of common cancers in the Atherosclerosis Risk in Communities study. *Cancer Causes Control.* 2013; 24: 2077-87.

28. **Pihoker C, Gilliam LK, Ellard S,** ***et al*.** SEARCH for Diabetes in Youth Study Group. Prevalence, characteristics and clinical diagnosis of maturity onset diabetes of the young due to mutations in HNF1A, HNF4A, and glucokinase: results from the SEARCH for Diabetes in Youth. *J Clin Endocrinol Metab.* 2013; 98: 4055-62.

29. **Estrada K, Aukrust I, Bjørkhaug L,** ***et al.*** Association of a low-frequency variant in HNF1A with type 2 diabetes in a Latino population. *JAMA.* 2014; 311: 2305-14.

30. **Tommasi S, Karm DL, Wu X,** ***et al*.** Methylation of homeobox genes is a frequent and early epigenetic event in breast cancer. *Breast Cancer Res*. 2009; 11: R14.

31. **Tjora E, Wathle G, Erchinger F,** ***et al*.** Exocrine pancreatic function in hepatocyte nuclear factor 1β-maturity-onset diabetes of the young (HNF1B-MODY) is only moderately reduced: compensatory hypersecretion from a hypoplastic pancreas. *Diabet Med*. 2013; 30: 946-55.

32. **Tare A, Lane JM, Cade BE, *et al*.** Sleep duration does not mediate or modify association of common genetic variants with type 2 diabetes. *Diabetologia.* 2014; 57: 339-46.

33. **Parry HM, Donnelly LA, Van Zuydam N, *et al*.** Wellcome Trust Case Control Consortium 2. Genetic variants predicting left ventricular hypertrophy in a diabetic population: a Go-DARTS study including meta-analysis. *Cardiovasc Diabetol*. 2013; 12: 109.

34. **Cao Y, Li Y, Kim J, *et al*.** Orally efficacious novel small molecule 6-chloro-6-deoxy-1, 2, 3, 4-tetra-O-galloyl-α-D-glucopyranose selectively and potently stimulates insulin receptor and alleviates diabetes. *J Mol Endocrinol.* 2013; 51: 15-26.

35. **Quan H, Tang H, Fang L, *et al*.** IGF1(CA)19 and IGFBP-3-202A/C gene polymorphism and cancer risk: a meta-analysis. *Cell Biochem Biophys.* 2014; 69: 169-78.

36. **Sbaraglini ML, Molinuevo MS, Sedlinsky C,** ***et al*.** Saxagliptin affects long-bone microarchitecture and decreases the osteogenic potential of bone marrow stromal cells. *Eur J Pharmacol.* 2014; 727: 8-14.

37. **Droste M, Domberg J, Buchfelder M,** ***et al*.** Therapy of acromegalic patients exacerbated by concomitant type 2 diabetes requires higher pegvisomant doses to normalise IGF1 levels. *Eur J Endocrinol.* 2014; 171: 59-68.

38. **Winder T, Giamas G, Wilson PM, *et al*.** Insulin-like growth factor receptor polymorphism defines clinical outcome in estrogen receptor-positive breast cancer patients treated with tamoxifen. *Pharmacogenomics J*. 2014; 14: 28-34.

39. **Alharbi KK, Khan IA, Abotalib Z, *et al*.** Insulin receptor substrate-1 (IRS-1) Gly927Arg: correlation with gestational diabetes mellitus in Saudi women. *Biomed Res Int.* 2014; 2014: 146495.

40. **Janes KA.** RUNX1 and its understudied role in breast cancer. *Cell Cycle.* 2011; 10: 3461-5.

41. **Smyth DJ, Howson JM, Payne F, *et al*.** Analysis of polymorphisms in 16 genes in type 1 diabetes that have been associated with other immune-mediated diseases. *BMC Med Genet.* 2006; 7: 20.

42. **Zhu Z, Tong X, Zhu Z, *et al*.** Development of GMDR-GPU for gene-gene interaction analysis and its application to WTCCC GWAS data for type 2 diabetes. *PLoS One*. 2013; 8: e61943.

43. **Blackman SM, Commander CW, Watson C,** ***et al*.** Genetic modifiers of cystic fibrosis-related diabetes. *Diabetes*. 2013; 62: 3627-35.

44. **Peng F, Hu D, Gu C, *et al*.** The relationship between five widely-evaluated variants in CDKN2A/B and CDKAL1 genes and the risk of type 2 diabetes: a meta-analysis. *Gene*. 2013; 531: 435-43.

45. **Hollier BG, Tinnirello AA, Werden SJ, *et al*.** FOXC2 expression links epithelial-mesenchymal transition and stem cell properties in breast cancer. *Cancer Res.* 2013; 73: 1981-92.

46. **Krings A, Rahman S, Huang S,** ***et al*.** Bone marrow fat has brown adipose tissue characteristics, which are attenuated with aging and diabetes. *Bone*. 2012; 50: 546-52.

47. **Håkansson J, Eliasson B, Smith U, *et al*.** Adipocyte mitochondrial genes and the forkhead factor FOXC2 are decreased in type 2 diabetes patients and normalized in response to rosiglitazone. *Diabetol Metab Syndr*. 2011; 3: 32.

48. **Tural D, Serdengecti S, Demirelli F, *et al*.** Clinical significance of p95HER2 overexpression, PTEN loss and PI3K expression in p185HER2-positive metastatic breast cancer patients treated with trastuzumab-based therapies. *Br J Cancer*. 2014; 110: 2996.

49. **Sezen SF, Lagoda G, Musicki B, *et al*.** Hydroxyl Fasudil, an Inhibitor of Rho Signaling, Improves Erectile Function in Diabetic Rats: A Role for Neuronal ROCK. *J Sex Med*. 2014.

50. **Wang L, Opland D, Tsai S, *et al*.** Pten deletion in RIP-Cre neurons protects against type 2 diabetes by activating the anti-inflammatory reflex. *Nat Med.* 2014; 20: 484-92.

51. **Arendt LM, McCready J, Keller PJ, *et al*.** Obesity promotes breast cancer by CCL2-mediated macrophage recruitment and angiogenesis. *Cancer Res.* 2013; 73: 6080-93.

52. **Simon MC, Bilan S, Nowotny B, *et al*.** Fatty acids modulate cytokine and chemokine secretion of stimulated human whole blood cultures in diabetes. *Clin Exp Immunol.* 2013; 172: 383-93.

53. **Kolattukudy PE, Niu J.** Inflammation, endoplasmic reticulum stress, autophagy, and the monocyte chemoattractant protein-1/CCR2 pathway. *Circ Res.* 2012; 110: 174-89.

54. **Sims AH, Finnon P, Miller CJ,** ***et al*.** TPD52 and NFKB1 gene expression levels correlate with G2 chromosomal radiosensitivity in lymphocytes of women with and at risk of hereditary breast cancer. *Int J Radiat Biol*. 2007; 83: 409-20.

55. **Katarina K, Daniela P, Peter N, *et al*.** HLA, NFKB1 and NFKBIA gene polymorphism profile in autoimmune diabetes mellitus patients. *Exp Clin Endocrinol Diabetes.* 2007; 115: 124-9.

56. **Stoynev N, Dimova, Rukova B,** ***et al*.** Gene expression in peripheral blood of patients with hypertension and patients with type 2 diabetes. *J Cardiovasc Med.* 2013.

57. **Alanazi M, Pathan AA, Shaik JP, *et al*.** The C allele of a synonymous SNP (rs1805414, Ala284Ala) in PARP1 is a risk factor for susceptibility to breast cancer in Saudi patients. *Asian Pac J Cancer Prev*. 2013; 14: 3051-6.

58. **Li B, Luo C, Chowdhury S, *et al*.** Parp1 deficient mice are protected from streptozotocin-induced diabetes but not caerulein-induced pancreatitis, independent of the induction of Reg family genes. *Regul Pept.* 2013; 186: 83-91.

59. **Narne P, Ponnaluri KC, Singh S,** ***et al*.** Relationship between NADPH oxidase p22phox C242T, PARP-1 Val762Ala polymorphisms, angiographically verified coronary artery disease and myocardial infarction in South Indian patients with type 2 diabetes mellitus. *Thromb Res.* 2012; 130: e259-65.

60. **Garcia-Murillas I, Sharpe R, Pearson A,** ***et al*.** An siRNA screen identifies the GNAS locus as a driver in 20q amplified breast cancer. *Oncogene.* 2014; 33: 2478-86.

61. **Tang H, Wei P, Duell EJ, *et al*.** Genes-environment interactions in obesity- and diabetes-associated pancreatic cancer: a GWAS data analysis. *Cancer Epidemiol Biomarkers Prev.* 2014; 23: 98-106.

62. **Celi FS, Coppotelli G, Chidakel A, *et al.*** The role of type 1 and type 2 5'-deiodinase in the pathophysiology of the 3,5,3'-triiodothyronine toxicosis of McCune-Albright syndrome. *J Clin Endocrinol Metab.* 2008; 93: 2383-9.

63. **Clements A, Robison K, Granai C, *et al*.** A case of Peutz-Jeghers syndrome with breast cancer, bilateral sex cord tumor with annular tubules, and adenoma malignum caused by STK11 gene mutation. *Int J Gynecol Cancer.* 2009; 19: 1591-4.

64. **Bassols J, Megia A, Soriano-Rodríguez P,** ***et al*.** A common gene variant in STK11 is associated with metabolic risk markers and diabetes during gestation. *Fertil Steril.* 2013; 100: 788-92.

65. **Keshavarz P, Inoue H, Nakamura N, *et al*.** Single nucleotide polymorphisms in genes encoding LKB1 (STK11), TORC2 (CRTC2) and AMPK alpha2-subunit (PRKAA2) and risk of type 2 diabetes. *Mol Genet Metab*. 2008; 93: 200-9.

66. **Gong Y, Wei W, Wu Y, *et al*.** Expression of androgen receptor in inflammatory breast cancer and its clinical relevance. *Cancer.* 2014; 120: 1775-9.

67. **El Ela MH, Azeem AA, Elbastawisy HI, *et al*.** Population genetic study of diabetes and diabetic retinopathy among Egyptians. *Bratisl Lek Listy.* 2012; 113: 595-8.

68. **Maruyama T, Onda K, Hayakawa M, *et al*.** Synthesis and evaluation of novel phenylethanolamine derivatives containing acetanilides as potent and selective beta3-adrenergic receptor agonists. *Chem Pharm Bull.* 2010; 58: 533-45.

69. **Shi H, Bevier M, Johansson R, *et al*.** Single nucleotide polymorphisms in the 20q13 amplicon genes in relation to breast cancer risk and clinical outcome. *Breast Cancer Res Treat.* 2011; 130: 905-16.

70. **Bellomo E, Massarotti A, Hogstrand C, *et al*.** Zinc ions modulate protein tyrosine phosphatase 1B activity. *Metallomics.* 2014.

71. **Bodhini D, Radha V, Ghosh S,** ***et al*.** Lack of association of PTPN1 gene polymorphisms with type 2 diabetes in south Indians. *J Genet.* 2011; 90: 323-6.

72. **Galluzzi L, De Santi M, Crinelli R,** ***et al*.** Induction of endoplasmic reticulum stress response by the indole-3-carbinol cyclic tetrameric derivative CTet in human breast cancer cell lines. *PLoS One*. 2012; 7: e43249.

73. **Price JA, Brewer CS, Howard TD, *et al.*** A physical map of the 20q12-q13.1 region associated with type 2 diabetes. *Genomics.* 1999; 62: 208-15.

74. **Bennett CE, Nsengimana J, Bostock JA, *et al*.** CCAAT/enhancer binding protein alpha, beta and delta gene variants: associations with obesity related phenotypes in the Leeds Family Study. *Diab Vasc Dis Res.* 2010; 7: 195-203.

75. **Ciftci R, Tas F, Yasasever CT,** ***et al*.** High serum transforming growth factor beta 1 (TGFB1) level predicts better survival in breast cancer. *Tumour Biol*. 2014.

76. **Nabrdalik K, Gumprecht J, Adamczyk P, *et al.*** Association of rs1800471 polymorphism of TGFB1 gene with chronic kidney disease occurrence and progression and hypertension appearance. *Arch Med Sci.* 2013; 9: 230-7.

77. **Fink LN, Oberbach A, Costford SR, *et al.*** Expression of anti-inflammatory macrophage genes within skeletal muscle correlates with insulin sensitivity in human obesity and type 2 diabetes. *Diabetologia*. 2013; 56: 1623-8.

78. **Tong Z, Yerramilli U, Surapaneni S,** ***et al*.** The interactions of lenalidomide with human uptake and efflux transporters and UDP-glucuronosyltransferase 1A1: lack of potential for drug-drug interactions. *Cancer Chemother Pharmacol*. 2014; 73: 869-74.

79. **Litim N, Labrie Y, Desjardins S, *et al.*** Polymorphic variations in the FANCA gene in high-risk non-BRCA1/2 breast cancer individuals from the French Canadian population. *Mol Oncol.* 2013; 7: 85-100.

80. **Li J, Sipple J, Maynard S, *et al*.** Fanconi anemia links reactive oxygen species to insulin resistance and obesity. *Antioxid Redox Signal.* 2012; 17: 1083-98.

81. **Thompson ER, Doyle MA, Ryland GL,** ***et al*.** Exome sequencing identifies rare deleterious mutations in DNA repair genes FANCC and BLM as potential breast cancer susceptibility alleles. *PLoS Genet*. 2012; 8: e1002894.

82. **Fagerholm R, Sprott K, Heikkinen T,** ***et al*.** Overabundant FANCD2, alone and combined with NQO1, is a sensitive marker of adverse prognosis in breast cancer. *Ann Oncol*. 2013; 24: 2780-5.

83. **Fei P, Yin J, Wang W.** New advances in the DNA damage response network of Fanconi anemia and BRCA proteins. FAAP95 replaces BRCA2 as the true FANCB protein. *Cell Cycle.* 2005; 4: 80-6.

84. **García MJ, Fernández V, Osorio A, *et al*.** Analysis of FANCB and FANCN/PALB2 fanconi anemia genes in BRCA1/2-negative Spanish breast cancer families. *Breast Cancer Res Treat*. 2009; 113: 545-51.

85. **Hanson RL, Bogardus C, Duggan D, *et al*.** A search for variants associated with young-onset type 2 diabetes in American Indians in a 100K genotyping array. *Diabetes*. 2007; 56: 3045-52.

86. **Bartsch DK, Langer P, Habbe N, *et al*.** Clinical and genetic analysis of 18 pancreatic carcinoma/melanoma-prone families. *Clin Genet.* 2010; 77: 333-41.

87. **Landi S.** Genetic predisposition and environmental risk factors to pancreatic cancer: A review of the literature. *Mutat Res*. 2009; 681: 299-307.

88. **Douglass S, Meeson AP, Overbeck-Zubrzycka D,** ***et al.*** Breast cancer metastasis: demonstration that FOXP3 regulates CXCR4 expression and the response to CXCL12. *J Pathol.* 2014.

89. **Baris S, Schulze I, Ozen A,** ***et al*.** Clinical Heterogeneity of Immunodysregulation, Polyendocrinopathy, Enteropathy, X-linked: Pulmonary Involvement as a Non-Classical Disease Manifestation. *J Clin Immunol*. 2014.

90. **Sun M, Song CX, Huang H, *et al.*** HMGA2/TET1/HOXA9 signaling pathway regulates breast cancer growth and metastasis. *Proc Natl Acad Sci U S A.* 2013; 110: 9920-5.

91. **Pegoraro S, Ros G, Piazza S, *et al*.** HMGA1 promotes metastatic processes in basal-like breast cancer regulating EMT and stemness. *Oncotarget.* 2013; 4: 1293-308.

92. **Semple RK.** From bending DNA to diabetes: the curious case of HMGA1. *J Biol*. 2009; 8: 64.

93. **Pullinger CR, Goldfine ID, Tanyolaç S,** ***et al*.** Evidence that an HMGA1 gene variant associates with type 2 diabetes, body mass index, and high-density lipoprotein cholesterol in a Hispanic-American population. *Metab Syndr Relat Disord.* 2014; 12: 25-30.

94. **Xiang H, Li H, Ge W,** ***et al*.** Association of CDKN1B gene polymorphisms with susceptibility to breast cancer: a meta-analysis. *Mol Biol Rep*. 2013; 40: 6371-7.

95. **Uchida T, Nakamura T, Hashimoto N, *et al*.** Deletion of Cdkn1b ameliorates hyperglycemia by maintaining compensatory hyperinsulinemia in diabetic mice. *Nat Med.* 2005; 11: 175-82.

96. **Liu LC, Su CH, Wang HC,** ***et al*.** Significant association of caveolin-1 (CAV1) genotypes with breast cancer in Taiwan. *Anticancer Res.* 2011; 31: 3511-5.

97. **Shi Y, Yang G, Yu J, *et al*.** Apolipoprotein CIII hyperactivates β cell CaV1 channels through SR-BI/β1 integrin-dependent coactivation of PKA and Src. *Cell Mol Life Sci.* 2014; 71: 1289-303.

98. **Petersen RK, Larsen SB, Jensen DM, *et al*.** PPARgamma-PGC-1alpha activity is determinant of alcohol related breast cancer. *Cancer Lett.* 2012; 315: 59-68.

99. **Yilmaz-Aydogan H, Kurnaz O, Kucukhuseyin O, *et al*.** Different effects of PPARA, PPARG and ApoE SNPs on serum lipids in patients with coronary heart disease based on the presence of diabetes. *Gene.* 2013; 523: 20-6.

100. **Kommoju UJ, Maruda J, Kadarkarai Samy S,** ***et al*.** Association of IRS1, CAPN10, and PPARG gene polymorphisms with type 2 diabetes mellitus in the high-risk population of Hyderabad, India. *J Diabetes.* 2014.

101. **Zhou LP, Luan H, Dong XH, *et al*.** Lack of association between LIG4 gene polymorphisms and the risk of breast cancer: a HuGE review and meta-analysis. *Asian Pac J Cancer Prev.* 2012; 13: 3417-22.

102. **Li D, Suzuki H, Liu B, *et al.*** DNA repair gene polymorphisms and risk of pancreatic cancer. *Clin Cancer Res*. 2009; 15: 740-6.

103. **Wang Z, Gu J, Nie W, *et al*.** Quantitative assessment of the association between three polymorphisms in FAS and FASL gene and breast cancer risk. *Tumour Biol.* 2014; 35: 3035-9.

104. **Rouq FA, Hammad D, Meo SA.** Protection of neuronal cell death against diabetes-induced apoptosis by Fas blocker ZB4. *J Int Med Res.* 2014.

105. **Nolsøe RL, Hamid YH, Pociot F,** ***et al.*** Association of a microsatellite in FASL to type II diabetes and of the FAS-670G>A genotype to insulin resistance. *Genes Immun.* 2006; 7: 316-21.

106. **Bianchi L, Canton C, Bini L,** ***et al*.** Protein profile changes in the human breast cancer cell line MCF-7 in response to SEL1L gene induction. *Proteomics*. 2005; 5: 2433-42.

107. **Li S, Francisco AB, Munroe RJ,** ***et al*.** SEL1L deficiency impairs growth and differentiation of pancreatic epithelial cells. *BMC Dev Biol*. 2010; 10: 19.

108. **Huang KT, Dobrovic A, Fox SB.** No evidence for promoter region methylation of the succinate dehydrogenase and fumarate hydratase tumour suppressor genes in breast cancer. *BMC Res Notes.* 2009; 2: 194.

109. **Murashima M, Kumar D, Doyle AM, *et al*.** Comparison of intradialytic blood pressure variability between conventional thrice-weekly hemodialysis and short daily hemodialysis. *Hemodial Int*. 2010; 14: 270-7.

110. **Seitz S, Korsching E, Weimer J,** ***et al*.** Genetic background of different cancer cell lines influences the gene set involved in chromosome 8 mediated breast tumor suppression. *Genes Chromosomes Cancer*. 2006; 45: 612-27.

111. **Iglesias-Platas I, Court F, Camprubi C, *et al*.** mprinting at the PLAGL1 domain is contained within a 70-kb CTCF/cohesin-mediated non-allelic chromatin loop. *Nucleic Acids Res*. 2013; 41: 2171-9.

112. **Gloyn AL, Mackay DJ, Weedon MN, *et al*.** Assessment of the role of common genetic variation in the transient neonatal diabetes mellitus (TNDM) region in type 2 diabetes: a comparative genomic and tagging single nucleotide polymorphism approach. *Diabetes.* 2006; 55: 2272-6.

113. **Garimella SV, Gehlhaus K, Dine JL, *et al*.** Identification of novel molecular regulators of Tumor Necrosis Factor-Related Apoptosis-Inducing Ligand (TRAIL)-induced apoptosis in breast cancer cells by RNAi screening. *Breast Cancer Res*. 2014; 16: R41.

114. **Herath TD, Darveau RP, Seneviratne CJ,** ***et al*.** Tetra- and penta-acylated lipid A structures of Porphyromonas gingivalis LPS differentially activate TLR4-mediated NF-κB signal transduction cascade and immuno-inflammatory response in human gingival fibroblasts. *PLoS One.* 2013; 8: e58496.

115. **Menzaghi C, Plengvidhya N, Ma X,** ***et al*.** Genetic variability in insulin action inhibitor Ikkbeta (IKBKB) does not play a major role in the development of type 2 diabetes. *J Clin Endocrinol Metab*. 2002; 87: 1894-7.

116. **Wallez Y, Riedl SJ, Pasquale EB.** Association of the breast cancer antiestrogen resistance protein 1 (BCAR1) and BCAR3 scaffolding proteins in cell signaling and antiestrogen resistance. *J Biol Chem*. 2014; 289: 10431-44.

117. **Magee TR, Ross MG, Wedekind L, *et al*.** Gestational diabetes mellitus alters apoptotic and inflammatory gene expression of trophobasts from human term placenta. *J Diabetes Complications*. 2014.

118. **Valentino R, Lupoli GA, Raciti GA, *et al*.** The PEA15 gene is overexpressed and related to insulin resistance in healthy first-degree relatives of patients with type 2 diabetes. *Diabetologia*. 2006; 49: 3058-66.

119. **Gasparini P, Fassan M, Cascione L, *et al*.** Androgen receptor status is a prognostic marker in non-basal triple negative breast cancers and determines novel therapeutic options. *PLoS One.* 2014; 9: e88525.

120. **Di Bartolo BA, Chan J, Bennett MR,** ***et al*.** TNF-related apoptosis-inducing ligand (TRAIL) protects against diabetes and atherosclerosis in Apoe ⁻/⁻ mice. *Diabetologia*. 2011; 54: 3157-67.

121. **Yu MY, Zhao PQ, Yan XH, *et al*.** Association between the TRAIL single nucleotide polymorphism rs1131580 and type 2 diabetes mellitus in a Han Chinese population. *Genet Mol Res*. 2013; 12: 3455-64.

122. **Huang-Doran I, Bicknell LS, Finucane FM, *et al*.** Majewski Osteodysplastic Primordial Dwarfism Study Group. Genetic defects in human pericentrin are associated with severe insulin resistance and diabetes. *Diabetes.* 2011; 60: 925-35.

**Supplemental Table 4**

**Diabetes, breast cancer, type 2** diabetes mellitus overlapping three modules of genes

| **Module** | **Gene** | **BC** | **DM** | **T2DM** |
| --- | --- | --- | --- | --- |
| M_b11.d12.2d11_ | pigl | - | - | - |
|  | agl | - | [1] | [2] |
|  | b3gnt1 | - | - | - |
|  | fut9 | - | - | - |
|  | st8sia4 | - | - | - |
|  | sult1a4 | - | - | - |
|  | ddost | - | [3] | - |
| M_b105.d89.2d68_ | lamp1 | [4] | [5] | - |
|  | ctsd | [6] | - | [7] |
|  | ctsb | [8] | - | - |
| M_b153.d141.2d99_ | ak3 | - | - | [9] |
|  | slc26a4 | - | [10] | - |
|  | cox7b | - | - | - |

1. **Desilles JP, Meseguer E, Labreuche J, *et al*.** Diabetes mellitus, admission glucose, and outcomes after stroke thrombolysis: a registry and systematic review. *Stroke.* 2013; 44: 1915-23.

2. **Chao PJ, Tsai JC, Chang DM,** ***et al*.** A case of acquired generalized lipodystrophy with cerebellar degeneration and type 2 diabetes mellitus. *Rev Diabet Stud.* 2004; 1: 193-7.

3. **Hoverfelt A, Sallinen R, Söderlund JM, *et al*.** innDiane Study Group. DDOST, PRKCSH and LGALS3, which encode AGE-receptors 1, 2 and 3, respectively, are not associated with diabetic nephropathy in type 1 diabetes. *Diabetologia.* 2010; 53: 1903-7.

4. **Abba MC, Fabris VT, Hu Y, *et al*.** Identification of novel amplification gene targets in mouse and human breast cancer at a syntenic cluster mapping to mouse ch8A1 and human ch13q34. *Cancer Res*. 2007; 67: 4104-12.

5. **Luppi P, Geng X, Cifarelli V, *et al*.** C-peptide is internalised in human endothelial and vascular smooth muscle cells via early endosomes. *Diabetologia.* 2009; 52: 2218-28.

6. **Mikulová V, Cabiňaková M, Janatková I, *et al*.** Detection of circulating tumor cells during follow-up of patients with early breast cancer: Clinical utility for monitoring of therapy efficacy. *Scand J Clin Lab Invest.* 2014; 74: 132-42.

7. **Perimenis P, Bouckenooghe T, Delplanque J, *et al*.** Placental antiangiogenic prolactin fragments are increased in human and rat maternal diabetes. *Biochim Biophys Acta.* 2014.

8. **Rafn B, Nielsen CF, Andersen SH,** ***et al*.** ErbB2-driven breast cancer cell invasion depends on a complex signaling network activating myeloid zinc finger-1-dependent cathepsin B expression*. Mol Cell.* 2012; 45: 764-76.

9. **Pullen TJ, Khan AM, Barton G,** ***et al*.** Identification of genes selectively disallowed in the pancreatic islet. *Islets*. 2010; 2: 89-95.

10. **Masindova I, Varga L, Stanik J, *et al*.** Molecular and hereditary mechanisms of sensorineural hearing loss with focus on selected endocrinopathies. *Endocr Regul.* 2012; 46: 167-86.
